# Directed Evolution of *Candidatus Methanomethylophilus alvus* Pyrrolysyl-tRNA Synthetase for the Genetic Incorporation of Two Different Noncanonical Amino Acids in One Protein

**DOI:** 10.1021/acsbiomedchemau.4c00028

**Published:** 2024-08-22

**Authors:** Chia-Chuan D. Cho, Waye Michelle Leeuwon, Wenshe Ray Liu

**Affiliations:** † Texas A&M Drug Discovery Center and Department of Chemistry, College of Arts and Sciences, Texas A&M University, College Station, Texas 77843, United States; ‡ Cancer Prevention and Research Institute of Texas, Austin, Texas 77843, United States; § Institute of Biosciences and Technology and Department of Translational Medical Sciences, School of Medicine, Texas A&M University, Houston, Texas 77030, United States; ∥ Department of Biochemistry and Biophysics, College of Agriculture and Life Sciences, Texas A&M University, College Station, Texas 77843, United States; ⊥ Department of Cell Biology and Genetics, School of Medicine, Texas A&M University, College Station, Texas 77843, United States; # Department of Pharmaceutical Sciences, Irma Lerma Rangel School of Pharmacy, Texas A&M University, College Station, Texas 77843, United States

**Keywords:** pyrrolysyl-tRNA synthetase, amber suppression, ochre suppression, noncanonical amino acid, directed evolution

## Abstract

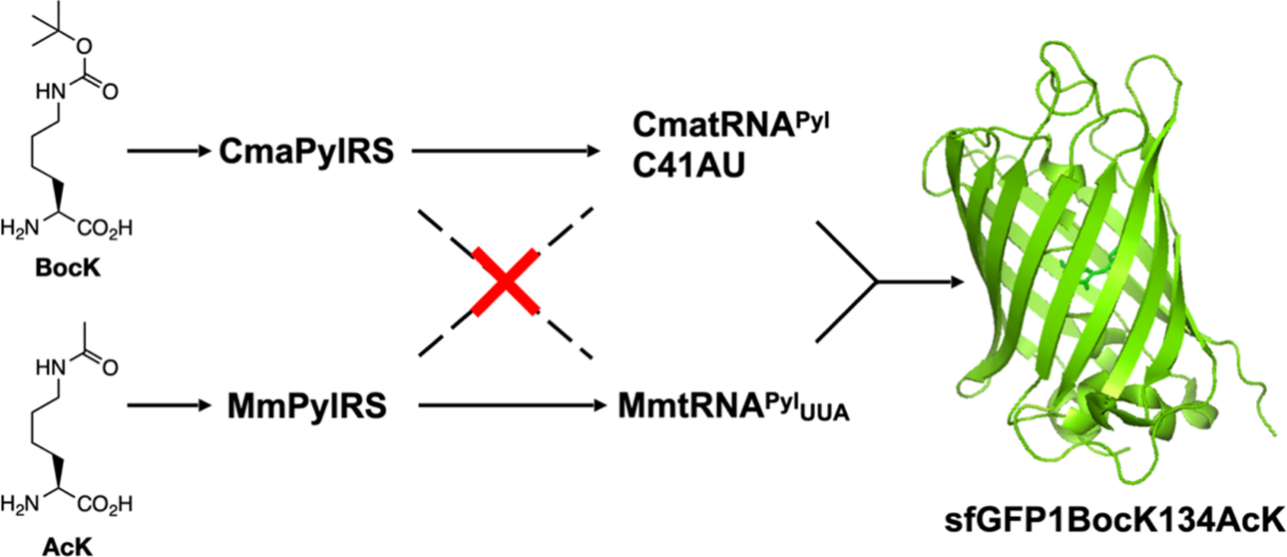

The genetic code expansion technique is a powerful chemical biology tool to install noncanonical amino acids (ncAAs) in proteins. As a key enzyme for this technique, pyrrolysyl-tRNA synthetase (PylRS), coupled with its cognate amber suppressor tRNA^Pyl^, has been engineered for the genetic incorporation of more than 200 ncAAs. Using PylRS clones from different archaeal origins, two ncAAs have also been genetically encoded in one protein. In this work, we show that the C41AU mutant of tRNA^Pyl^ from *Candidatus Methanomethylophilus alvus* (CmatRNA^Pyl^) is catalytically inert toward PylRS from *Methanosarcina mazei* (MmPylRS) but has weak activity toward PylRS from *Ca. M. alvus* (CmaPylRS). To improve the catalytic efficiency of CmaPylRS toward CmatRNA^Pyl^-C41AU, we conducted a directed evolution of CMaPylRS by randomizing its coding sequence, followed by the screening of active mutant clones. After three rounds of randomization and screening, we identified 4 mutations, Y16F/N57D/E161G/N182I, that improve the catalytic efficiency of CMaPylRS toward CMatRNA^Pyl^-C41AU. This new clone, named R3–14, coupling with CmatRNA^Pyl^-C41AU to recognize an amber codon, has been successfully used together with an evolved MmPylRS clone, coupling with a mutant *M. mazei* tRNA^Pyl^ to recognize an ochre codon, to genetically incorporate two different ncAAs, *N*^ε^-(*t*-butoxycarbonyl)-lysine and *N*^ε^-acetyl-lysine, into one model protein.

The discovery of the pyrrolysine (Pyl) incorporation system that utilizes the amber stop codon to code Pyl, the 22nd proteinogenic amino acid pyrrolysine, has opened many possibilities for protein engineering.^1,2^ The system includes a unique pyrrolysyl-tRNA synthetase (PylRS)-amber suppressor tRNA^Pyl^ pair that interacts with each other but not with any other aminoacyl-tRNA synthetase (aaRS) or tRNA in cells. The PylRS-tRNA^Pyl^ pair is also only catalytically active toward Pyl and shows no activity toward the canonical 20 proteinogenic amino acids. To date, numerous PylRS-tRNA^Pyl^ homologues have been identified in different methanogenic archaeal and certain bacterial strains. Clones from *Methanosarcina bakeri* and *Methanosarcina mazei* are the most used to conduct the noncanonical amino acid (ncAA) mutagenesis in different hosts including bacteria, yeast, and mammalian cells.^3,4^ PylRS has a large active site that mainly involves the van der Waals interactions to bind Pyl. This large active site leads to high substrate promiscuity for the native enzyme. The lack of unique interactions with Pyl has also made the enzyme amenable for engineering to bind novel ncAAs.^5^ Engineering PylRS from *M. bakeri* and *M. mazei* (named MbPylRS and MmPylRS, respectively) has allowed the genetic encoding of more than 200 different ncAAs. Both MbPylRS and MmPylRS are two-domain enzymes.^6,7^ Their N-terminus domain (NTD) is a tRNA binding domain, and their C-terminus domain (CTD) is a catalytic domain.^8^ X-ray crystallography analyses have indicated that both NTD and CTD do not interact with the anticodon region of tRNA^Pyl^.^9,10^ This unique feature has made it possible to mutate the anticodon of tRNA^Pyl^ to recognize opal, ochre, and even four-base codons for their reassignment to code ncAAs.^11−13^ Using this unique feature and coupled with aaRS-tRNA pairs from other origins, two different ncAAs have been genetically encoded using two stop codons or one stop and one four-base codon.^14,15^ Built upon these early works, additional efforts have been made by engineering the cellular systems to incorporate up to 4 ncAAs into a single protein, contributed by the combined efforts from Chin, Söll, Chatterjee, Schultz, and co-workers.^16−24^

The ability of different codon recognition potentially allows for coding two different ncAAs using two PylRS-tRNA^Pyl^ pairs that do not cross-interact with each other. This was not explored until the discovery of the PylRS-tRNA^Pyl^ pair from *Candidatus Methanomethylophilus alvus*.^25^ PylRS from *Ca. M. alvus* (CmaPylRS) contains only a CTD domain, indicating that its recognition of tRNA^Pyl^ from *Ca. M. alvus* (CmatRNA^Pyl^) is different from the other two-domain PylRSs. By mutating CmatRNA^Pyl^ to generate orthogonality toward MmPylRS, Willis et al. were able to show that two ncAAs could be coded in one protein by amber and four-base codon, respectively, using two PylRS-tRNA^Pyl^ pairs from *M. mazei* and *Ca. M. alvus*.^26^ However, CmaPylRS has a relatively low activity toward a mutant CmatRNA^Pyl^.^27^ We aimed to improve this catalytic efficiency through directed evolution for the improved incorporation of two different ncAAs that are both lysine derivatives into one protein. In addition, two stop codons, amber and ochre, are used for the incorporation of two ncAAs for the simplicity of the method.

MmPylRS and CmaPylRS (gene names: MmPylS and CamPylS) share only 41% sequence identity (Figure 1A), and CmaPylRS completely lacks an NTD domain, making them ideal for testing orthogonal incorporation of two different ncAAs. The two tRNA^Pyl^’s, CmatRNA^Pyl^ and *M. mazei* tRNA^Pyl^ (MmtRNA^Pyl^) with gene names CmaPylT and MmPylT, respectively, also show large sequence variations at the acceptor stem, TψC loop, D loop, and anticodon stem (Figure 1B). Both PylRS’s naturally recognize *N*^ε^-(*t*-butoxycarbonyl)-lysine (BocK, Figure 1C). We used this ncAA together with PylRS and tRNA^Pyl^ genes from *M. mazei* and *Ca. M. alvus* to test amber suppression efficiency and cross-reactivity between PylRS and tRNA^Pyl^ from the two different origins. We constructed four plasmids coding MmPylRS-MmtRNA^Pyl^, MmPylRS-CmatRNA^Pyl^, CmaPylRS-MmtRNA^Pyl^, and CmaPylRS-CmatRNA^Pyl^ and used them to transform Top10 *Escherichia coli* cells that harbored an expression vector coding for a superfolder green fluorescent protein (sfGFP) gene with an amber stop codon mutation at its D134 coding position. Transformed cells were grown in a 2YT medium supplemented with 1 mM BocK and 0.2% arabinose to trigger the expression of full-length sfGFP. The expression of full-length sfGFP was then quantified by fluorescence (Ex/Em: 485/510 nm) measured by a Synergy Neo2 plate reader. Results are presented in Figure 1D. As shown, MmPylRS recognizes both MmtRNA^Pyl^ and CmatRNA^Pyl^ for the genetic incorporation of BocK at the amber codon. The MmPylRS-CmatRNA^Pyl^ pair exhibited almost twice as good overall BocK incorporation efficiency than the MmPylRS-CmaPylRS pair. On the contrary, CmaPylRS recognizes only CmatRNA^Pyl^ for the genetic incorporation of BocK at amber codon and showed negligent BocK incorporation at amber codon when it was used together with MmtRNA^Pyl^. These results indicate that CmaPylRS is orthogonal toward MmtRNA^Pyl^ but that MmPylRS cross-reacts with CmatRNA^Pyl^. Two plasmids coding MmPylRS-MmtRNA^Pyl^ or CmaPylRS-CmatRNA^Pyl^ with the tRNA anticodon mutated to UUA to recognize the ochre codon were also constructed. They were used to transform Top10 *E. coli* cells containing a plasmid coding sfGFP with a corresponding ochre mutation at the D134 position. Cells were then grown in the presence of 1 mM BocK and 0.2% arabinose. As shown in Figure S3, both MmPylRS-MmtRNA_UUA_^Pyl^ and CmaPylRS-CmatRNA_UUA_^Pyl^ pairs mediate the genetic incorporation of BocK at the ochre codon with reduced BocK incorporation compared to the amber codon. Therefore, both pairs can use the ochre codon as an alternative codon to code ncAAs, making it possible to use these two pairs and two stop codons, one amber and the other ochre, to code two different ncAAs. We chose the MmPylRS-MmtRNA_UUA_^Pyl^ pair for the ochre suppression due to its better suppression level shown in Figure S3.

**Figure 1:**
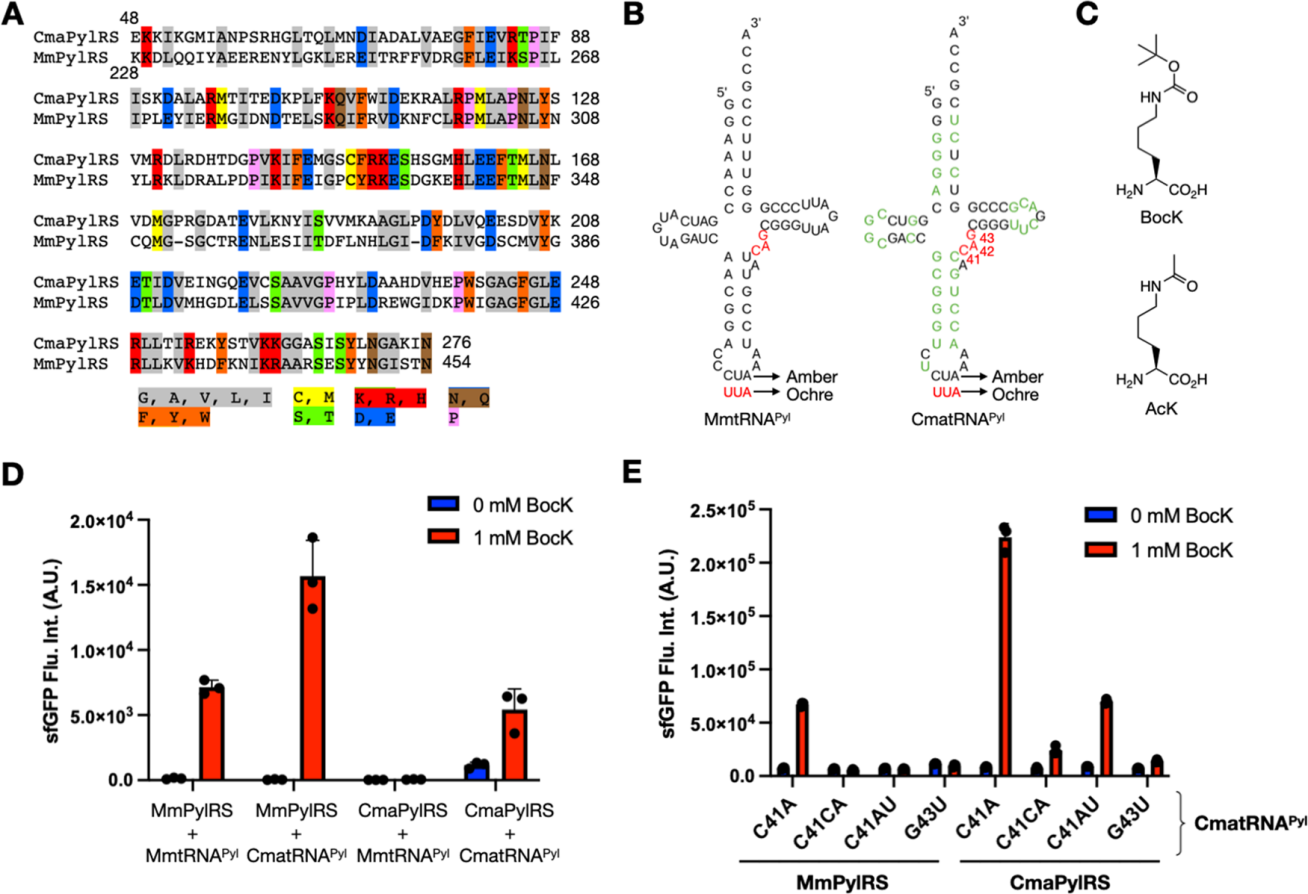
(A) Amino acid sequence alignment between *Ca. M. alvus* PylRS (CmaPylRS) and *M. mazei* PylRS (MmPylRS) at their catalytic domain. Identical and homologous residues are highlighted according to amino acid color codes shown at the bottom of the figure panel. (B) The secondary structures of *M. mazei* tRNA^Pyl^ (MmtRNA^Pyl^) and *Ca. M. alvus* tRNA^Pyl^ (CmatRNA^Pyl^). The variable arm and numbering of its nucleotides are indicated in CmatRNA^Pyl^. The CUA anticodon, its mutation to the UUA anticodon, and their recognized codons are indicated as well. Nucleotides in CmatRNA^Pyl^ different from those in MmtRNA^Pyl^ are colored in green. Nucleotides in the variable arm for both tRNAs are colored red. (C) Chemical structures of *N*^ε^-*tert*-butyloxycarbonyl-l-lysine (BocK) and *N*^ε^-acetyl-l-lysine (AcK). (D) The cross-recognition tests between PylRS and tRNA^Pyl^ from *M. mazei* and *Ca. M. alvus*. (E) CmatRNA^Pyl^ mutants and their recognition by MmPylRS and CmaPylRS for the genetic incorporation of BocK at the amber codon.

To use CmaPylRS-CmatRNA^Pyl^ and MmPylRS-MmtRNA_UUA_^Pyl^ pairs for the orthogonal incorporation of two different ncAAs at amber and ochre codons, respectively, the cross-reactivity of MmPylRS toward CmatRNA^Pyl^ has to be resolved. It has been shown that the two-domain PylRS enzymes recognize the variable arm in their cognate tRNA^Pyl^s for binding.^10,28^ CmatRNA^Pyl^ has a variable arm the same as the one in MmtRNA^Pyl^. This likely contributes to its recognition by MmPylRS.^10^ As shown in Figure 1E, mutating C41 in this variable arm to dinucleotides CA and AU or G43 to U led to inactive recognition of the corresponding CmatRNA^Pyl^ mutants by MmPylRS. MmPylRS still recognizes the C41A mutant of CmatRNA^Pyl^, although its activity is significantly weaker than that of CmaPylRS toward this mutant. Except for the G43U mutant, the other three CmatRNA^Pyl^ mutants can still be recognized by CmaPylRS. These results corroborate what has been reported by Willis et al.^26^ Since both C41CA and C41AU mutants of CmatRNA^Pyl^ are not recognized by MmPylRS and still active toward CmaPylRS, their pairs with CmaPylRS can be potentially used together with MmPylRS-MmtRNA_UUA_^Pyl^ for the orthogonal incorporation of two different ncAAs at amber and ochre codons, respectively. Since CmaPylRS is more reactive toward CmatRNA^Pyl^-C41AU than CmatRNA^Pyl^-C41CA, we chose CmatRNA^Pyl^-C41AU to move forward.

Compared with wild-type CmatRNA^Pyl^, the C41AU mutant is much less reactive toward CmaPylRS (Figure S5). To improve the reactivity of CmaPylRS toward CmatRNA^Pyl^-C41AU, we then conducted a directed evolution. Two plasmids with basic structures shown in Figures 2A and S6 were used for this directed evolution approach. The first pBK-CmaPylRS plasmid encodes the CmaPylRS gene (CmaPylS is the gene name) undergoing randomization. The CmaPylS gene is under the control of glutamine synthetase (Gln *S*) promoter that continuously drives the expression of CmaPylRS. The second pY^+^-CmatRNA^Pyl^-C41AU plasmid encodes a CmatRNA^Pyl^-C41AU gene (CmaPylT-C41AU as its gene name), a chloramphenicol acetyltransferase (Chl^R^) gene with two amber mutations at N2 and D44 coding positions, a T7 RNA polymerase (T7RNAP) gene that contains two amber mutations at M1 and Q107 coding positions and an MTMITVH leading peptide, and an ultraviolet light-excited green fluorescence protein (GFP_UV_) gene under control of a T7/Lac promoter. The encoded Chl^R^ gene provides a survival check to allow only cells with strong amber suppression to survive in the presence of chloramphenicol. The T7RNAP and GFP_UV_ genes work together to allow cells with strong amber suppression to exhibit strong green fluorescence under UV light, which can be visually detected. We confirmed that Top10 *E. coli* cells with only the pY^+^-CmatRNA^Pyl^-C41AU plasmid alone provide neither survival in the presence of chloramphenicol nor the expression of GFP_UV_ regardless of whether or not 1 mM BocK was provided in the 2YT medium. When Top10 *E. coli* cells were transformed with both pBK-CmaPylRS and pY^+^-CmatRNA^Pyl^-C41AU, the transformed cells survived in the presence of chloramphenicol and expressed GFP_UV_ only when 1 mM BocK was provided (Figure S7). These results demonstrated that CmatRNA^Pyl^-C41AU is orthogonal toward *E. coli* aaRSs and that CmaPylRS is able to aminoacylate CmatRNA^Pyl^-C41AU with BocK for amber suppression. To search for CmaPylRS mutants that are more catalytically efficient toward CmatRNA^Pyl^-C41AU, a randomized CMaPylS gene library was created using error-prone PCR (GenMorph II, Agilent 200550) to amplify the whole pBK-CmaPylRS. The linearized pBK-CmaPylRS was cyclized through the *Pst*I site (Figure 2B). The afforded plasmid library was transformed into Top10 *E. coli* cells harboring pY^+^-CmatRNA^Pyl^-C41AU. Cells were first grown in the 2YT medium supplemented with 0.1 mM BocK and 175 μg/mL of chloramphenicol to remove inactive or weakly active CmaPylRS mutant clones. Survived cells were then collected and plated on LB agar containing BocK (0.1 mM), 1 mM IPTG, and 0.2% arabinose for fluorescence-based screening (Figure 2C). 38 colonies with strong fluorescence were collected and grown in a 1 mL 2YT medium and then spotted with 10 μL on LB agar plates supplemented with varied concentrations of BocK (0 and 0.1 mM). Four colonies were identified with improved fluorescence compared to control cells transformed with the original pBK-CmaPylRS and pY^+^-CmatRNA^Pyl^-C41AU plasmids. They were subsequently sequenced to identify mutations and named as R1–6 (Y16F/N124I/E161G), R1–8 (Y21H/Q107R/I184V/L193M), R1–3 (A76D/M188T/E201V/E202V), and R1–4 (L63V/K189N). When spotted on LB agar in the presence of 0.1 mM BocK, the R1–6 and R1–8 clones exhibited much better GFP_UV_ expression compared to wild-type CmaPylRS (Figure 2C). We then followed a gene shuffling strategy to shred mutations in all four clones. The afforded DNA library was then cloned into the pBK plasmid through *Nde*I and *Pst*I. The afforded plasmid library was transformed into Top10 *E. coli* cells harboring pY^+^-CmatRNA^Pyl^-C41AU. However, compared to cells containing pBK-R1–6 or pBK-R1–8 and pY^+^-CmatRNA^Pyl^-C41AU, all transformed cells showed reduced fluorescence. R1–6 and R1–8 clones were then grown in the presence of 1 mM BocK and compared to the wild type in cells harboring pBK and pY^+^ plasmids. Both clones expressed better GFP_UV_ than wild type in the presence of 1 mM BocK (Figure 2D). Compared to the 0.1 mM BocK condition, the GFP_UV_ expression differences were less dramatic, likely due to the saturation of enzymes by BocK at this condition. We chose R1–6 to move forward to the next round of evolution and selection due to the few mutation sites identified.

**Figure 2:**
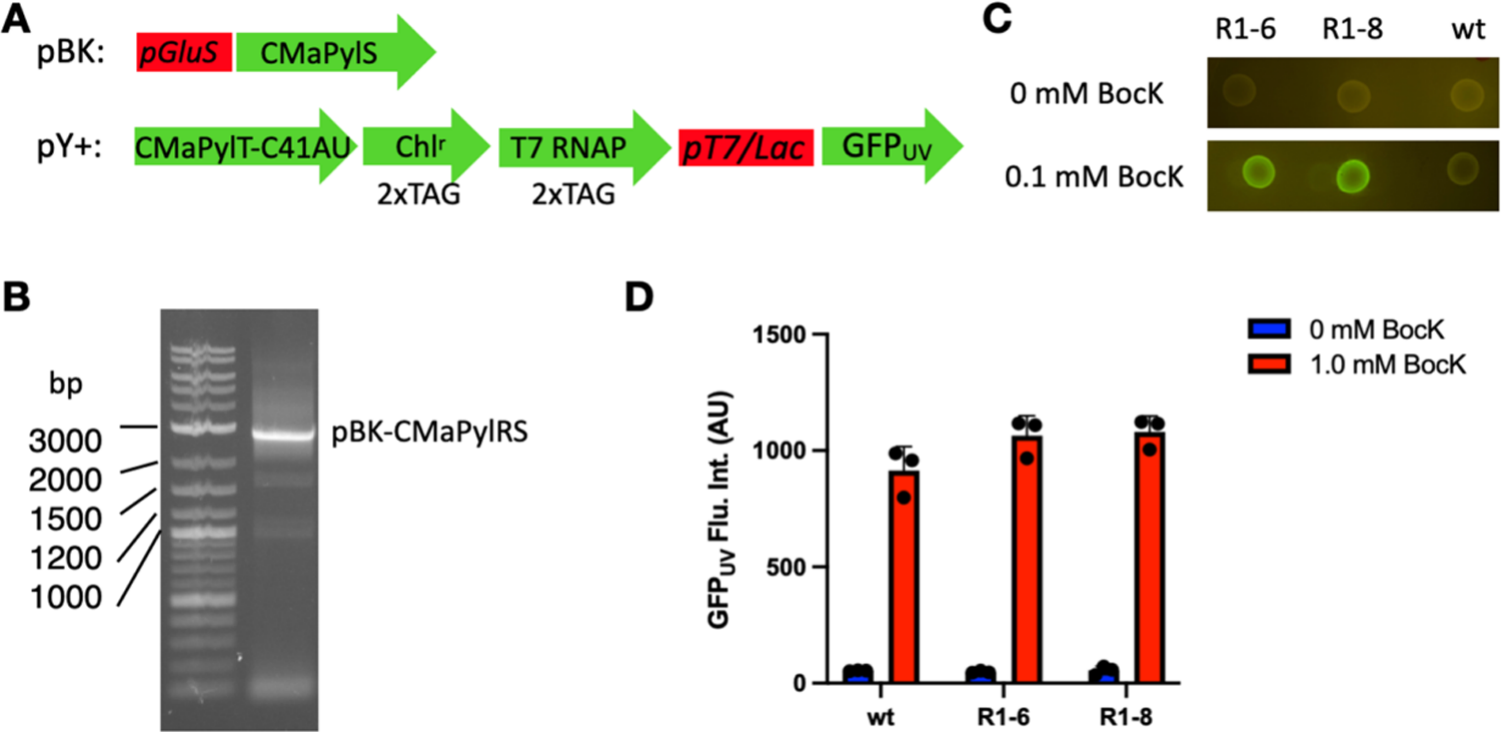
(A) Two plasmids used for the directed evolution and selection procedures. (B) GeneMorph II-randomized whole-plasmid pBK-CmaPylRS detected by the agarose gel electrophoresis. (C) On-plate comparison of R1–6 and R1–8 to the wild-type (wt) CmaPylRS in driving T7 promoter-controlled GFP_UV_ expression in cells containing pBK and pY^+^ plasmids and in the presence of 0.1 mM BocK. (D) In-medium comparison of R1–6 and R1–8 to wt CmaPylRS in driving T7 promoter-controlled GFP_UV_ expression in cells containing pBK and pY^+^ plasmids and in the presence of 1.0 mM BocK. Cells were also grown in the absence of BocK as negative controls.

We then proceeded to use R1–6 to conduct a second round of mutagenesis and screening. pBK-R1–6 was first sequence-randomized using the GenMorph II kit and cyclized through the *Pst*I site. The afforded plasmid library was then used to transform *E. coli* Top10 cells containing the PY^+^-CmatRNA^Pyl^-C41AU plasmid. Cells were then grown in 2YT medium supplemented with 0.1 mM BocK and 175 μg/mL of chloramphenicol. The surviving cells were plated on LB agar containing 0.1 mM BocK, 0.2% arabinose, and 1 mM IPTG for fluorescence screening. 40 colonies that exhibited fluorescence were then picked and plated on LB agar plates supplemented with 0.1 mM BocK for comparison with Top10 *E. coli* cells coding the wild-type CmaPylRS clone. Six clones that showed better fluorescence at 0.1 mM BocK were then grown in the presence of 0.5 and 1 mM BocK. Results are shown in Figure 3A. They all displayed better GFP_UV_ expressions. What was intriguing was the sequencing results. Compared to R1–6, both R2–2 and R2–7 clones mutated N124I back to N. In addition, R2–2 has a K181M mutation, while R2–7 has an N182I mutation. It is very interesting to observe the reversion of N124I back to N in R2–2 and R2–7 clones. Given the vast number of mutations that could be introduced into R1–6, there was a very rare chance of this particular site to be converted back. The screening results of R2–2 and R2–7 indicate that N124 could be a critical residue for the enzyme to maintain folding stability or strong binding to CmatRNA^Pyl^-C41AU. We also cloned 6 identified new CmaPylRS clones to the pEVOL template vector containing a gene coding CmatRNA^Pyl^-C41AU. The afforded vectors were used separately to transform Top10 *E. coli* cells containing the pBAD-sfGFP134TAG plasmid. The transformed cells were grown in the presence of 0.5 or 1 mM BocK to directly observe the genetic incorporation of BocK at the D134TAG mutation site in sfGFP and compared to cells coding wild-type CmaPylRS. The results showed R2–7 as a better clone than the wild type, R1–6, and all other clones identified from the second round of mutagenesis and screening (Figure 3B).

**Figure 3:**
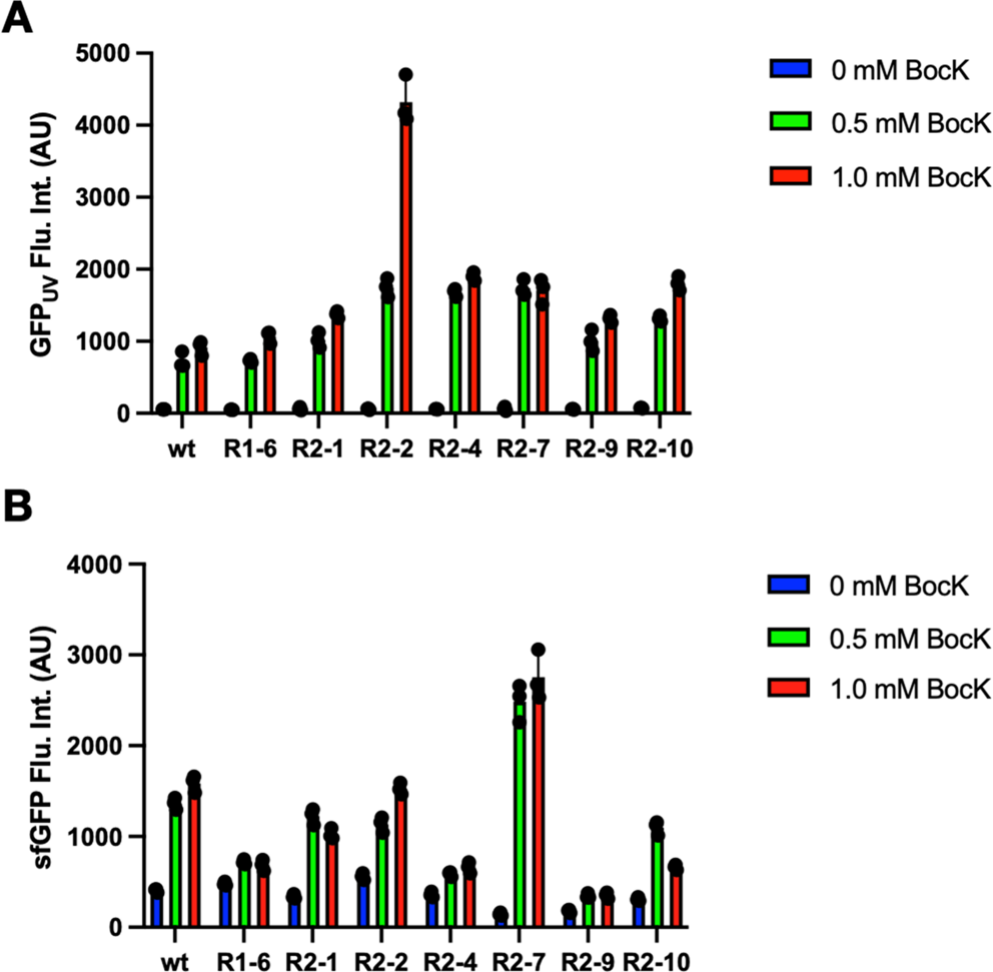
Comparison between different CmaPylRS clones. (A) Top10 cells harboring pBK-CmaPylRS and pY^+^ plasmids were grown in the presence of 0, 0.5, or 1 mM BocK and their expressed GFP_UV_ was recorded and plotted. (B) Top10 cells harboring pEVOL-CmaPylRS and pBAD-sfGFP134TAG were grown in the presence of 0, 0.5, and 1 mM BoK and their expressed sfGFP was recorded and plotted.

Encouraged by the results from the first two rounds of mutagenesis and screening, we proceeded to use R2–7 as a template to conduct the third round of error-prone mutagenesis using the Agilent GenMorph II kit. In this round, we directly cloned the afforded randomized R2–7 DNA into the pEVOL template through the *Spe*I and *Sal*I restriction sites. The afforded pEVOL plasmid library was used to transform Top10 *E. coli* cells containing the pBAD-sfGFP134TAG plasmid. The transformed cells were then plated on LB agar plates containing 0.2% arabinose and 0.1 mM BocK. Colonies with strong fluorescence were collected and spotted, as previously described. Strongly fluorescent clones were then grown in 2YT medium supplemented with 1 mM BocK and compared to the wild-type clone. As depicted in Figure 4A, R3–9 and R3–14 showed a clearly improved BocK incorporation at amber codon compared to wild-type and R2–7 clones when they were coupled with CmatRNA^Pyl^-C41AU. DNA sequencing showed that R3–9 has no new mutation compared to R2–7, while R3–14 contains an extra N57D mutation. The difference in the sfGFP expression for R2–7 and R3–9 clones is likely due to mutagenesis introduced in other parts of pEVOL or pBAD plasmids in R3–9, although different vector copy numbers in the two cells are also likely. We did not explore further about this difference since it is not directly related to the goal of finding more active CmaPylRS clones toward CmatRNA^Pyl^-C41AU. R3–9 and R3–14, coupled with CmatRNA^Pyl^-C41AU, were then advanced to the next step to test for their capability in mediating the genetic incorporation of BocK and AcK at amber and ochre codons, separately by working together with the MmAcKRS1-MmtRNA_UUA_^Pyl^ pair. MmAcKRS1 is a MmPylRS mutant that was previously evolved for the genetic incorporation of AcK.^29^

**Figure 4:**
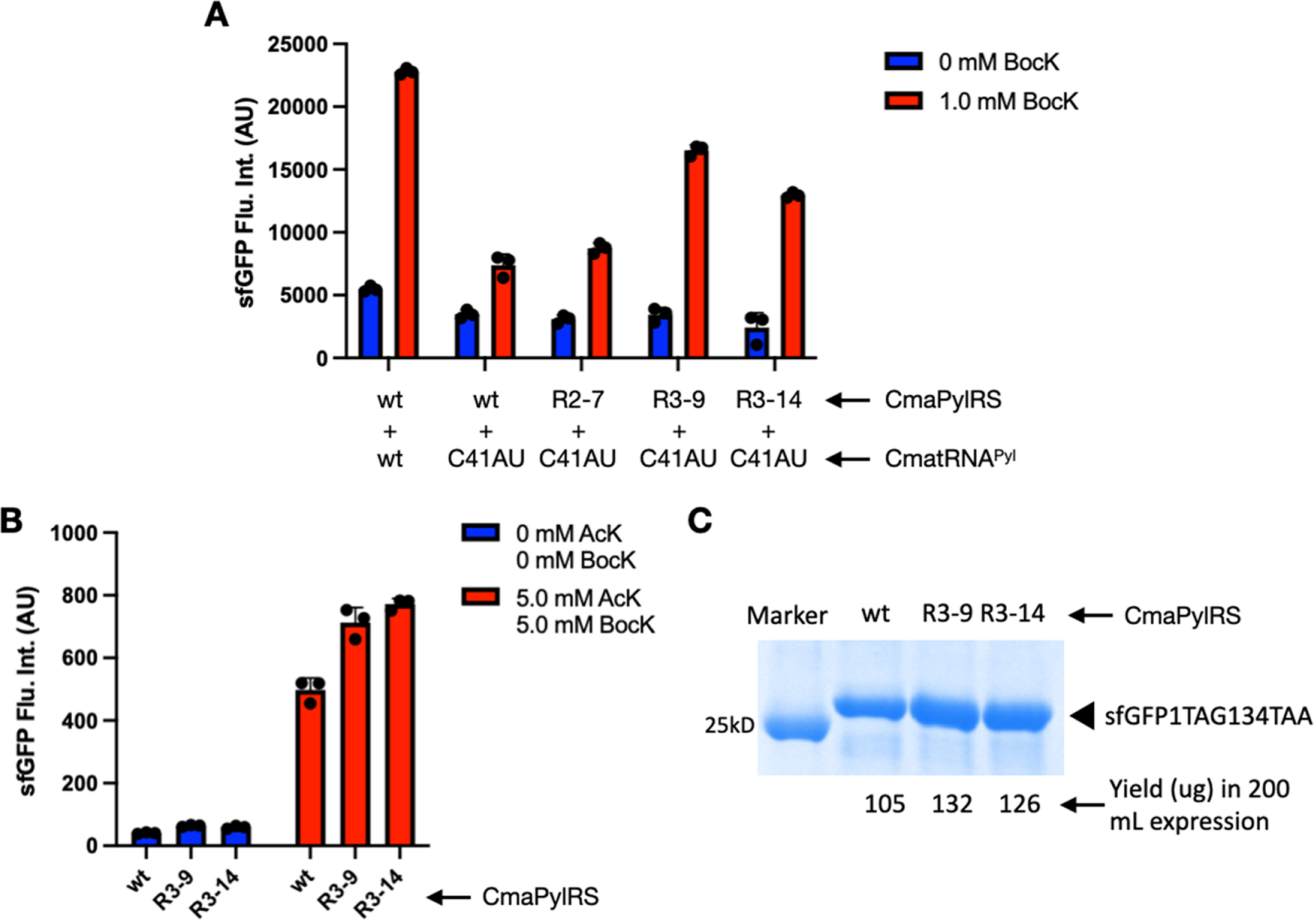
Round 3 selection results and the genetic incorporation of two different ncAAs into one protein. (A) Different CmaPylRS variants driving the genetic incorporation of BocK directly at the D134TAG mutation site in sfGFP by coupling with the wild-type or C41AU mutant of CmatRNA^Pyl^. (B) Double incorporation of BocK and AcK at M1TAG and D134TAA mutation sites, respectively, in the sfGFP gene using wild-type or evolved CmaPylRS-CmatRNA^Pyl^-C41AU and MmAcKRS1-MmtRNA_UUA_^Pyl^ pairs in Top10 cells. (C) The SDS-PAGE analysis of purified full-length sfGFP expressed in panel (B) and their quantified yields.

To test the genetic incorporation of BocK and AcK at the amber and ochre codons, respectively, two plasmids were used. The first plasmid is the pEVOL vector containing genes coding MmAcKRS1 and MmtRNA_UUA_^Pyl^ for ochre suppression. The second plasmid is based on the pBAD vector in which an sfGFP gene containing a TAG mutation at M1, a TAA mutation at D134, and an additional N-terminal Met-Ala was introduced. Genes coding CmaPyl^Pyl^-C41AU and wild-type, R3–9, or R3–14 CmaPylRS were also cloned into this pBAD vector. Cells transformed with the two vectors were then grown in two conditions, the first without providing any ncAAs and the second with 5 mM BocK and 5 mM AcK. The full-length sfGFP expression quantified by the detected fluorescence is shown in Figure 4B. When no ncAAs were provided, there was negligible full-length sfGFP expressed. As a control for orthogonality between AcK and BocK, we also carried out expression with 5 mM AcK and 10 mM BocK, respectively; there was negligible full-length sfGFP expressed for both conditions. Adding two ncAAs triggered full-length sfGFP expression. And the R3–9 and R3–14 clones are better than the wild-type CmaPylRS to drive the full-length sfGFP expression. The expressed sfGFP was then purified and quantified by the A660 nm protein assay (ThermoFisher, 22662). The R3–9 and R3–14 clones provided about 30% more sfGFP expression than the wild-type CmaPylRS (Figure 4C), matching the observation made in Figure 4B. All three purified proteins were also characterized by using the electrospray ionization mass spectrometry (ESI-MS) technique. As shown in Figure S10, they all showed the highest peak with a molecular weight at 28,049 or 28,050 Da that matches exactly the calculated molecular weight of full-length sfGFP with BocK incorporated at M1 and AcK incorporated at D134 but with the first methionine hydrolyzed. All three ESI-MS spectra showed the second-highest peak at 27,991 or 27,992 Da. These peaks matched full-length sfGFP with AcK incorporated at both M1 and D134 positions. Wild-type PylRS is known to have substrate promiscuity and recognize AcK weakly. This peak is likely due to the recognition of AcK by CmaPylRS clones, leading to AcK incorporation at the M1 position, although we cannot rule out the possibility of the wobble recognition of the UAG stop codon by MmtRNA_UUA_^Pyl^. In the first situation, the orthogonality of PylRS clones toward different ncAAs needed to be improved to avoid cross-reactivity. In the second situation, other codons such as four-base codons might be applied to resolve the cross-reactivity. The low expression level shown in Figure 4C might be improved by using RF1-knockout cell strains that have demonstrated with high amber suppression efficiencies.^30,31^

In summary, we tested the orthogonality of PylRS-tRNA^Pyl^ from two origins, *M. mazei* and *Ca. M. alvus*, toward each other and confirmed that CmaPylRS is orthogonal toward MmtRNA^Pyl^ but MmPylRS has a high catalytic efficiency toward CmatRNA^Pyl^. The introduction of a C41AU mutation completely blocks the recognition of CmatRNA^Pyl^ by MmPylRS, corroborating results from a previous report. Both MmtRNA^Pyl^ and CmatRNA^Pyl^ can be mutated at their anticodon to recognize ochre codon for coding an ncAA. CmatRNA^Pyl^-C41AU displays a reduced activity toward CmaPylRS compared to the wild type. To improve this activity, we conducted three rounds of mutagenesis and screening of CmaPylRS clones. These efforts have successfully led to the discovery of R3–9 and R3–14 clones with mutations such as Y16F/E161G/N182I and N57D/Y16F/E161G/N182I, respectively, which show better amber suppression by working together with CmatRNA^Pyl^-C41AU and with BocK as a substrate. The identified clones were then successfully applied to combining with CmatRNA^Pyl^ and the MmAcKRS1-MmtRNA_UUA_^Pyl^ to demonstrate the genetic incorporation of two different ncAAs, BocK and AcK, at amber and ochre mutation sites, respectively, in an sfGFP gene. We believe that these evolved CmaPylRS clones will be useful in future applications for the genetic incorporation of two ncAAs in one protein. Additional work that tests the potential recognition of the UAG codon by an ochre suppression through wobble base pairing might also be conducted.

## Materials and Methods

### Construction of Plasmids to Test Orthogonality

pEVOL-MmPylS and pBAD-sfGFP134TAG were obtained from previous members. MmPylS/MmPylT was removed from the pEVOL plasmid by *Spe*I and *Xho*I digestion. CmaPylS/CmaPylT genes were ordered from IDT and amplified with CmaPylRS-for and CmaPylRS-rev, which contained *Spe*I and *Xho*I restriction sites for cloning. The restriction-digested fragment was ligated to the pEVOL backbone through *Spe*I and *Xho*I sites. The product was then used to transform Top10 *E. coli* chemically competent cells. The single colonies were collected and sequence-verified.

pEVOL-MmPylS/CmaPylT and pEVOL-CmaPylS/MmPylT were generated by *Spe*I and *Sal*I restriction digests to pEVOL-MmPylS/MmPylT and pEVOL-CmaPylS/CmaPylT. The PylS gene fragment and pEVOL backbone were swapped to afford the impaired PylS/PylT. It was then used to transform Top10 *E. coli* competent cell and sequence-verified.

### Quantification of Amber Suppression

pEVOL-MmPylS/MmPylT, pEVOL-MmPylS-CmaPylT, pEVOL-CmaPylS-MmPylT, and pEVOL-CmaPylS-CmaPylT were used to transform Top10 *E. coli* competent cells harboring pBAD-sfGFP134TAG. Single colonies were inoculated to 5 mL of 2YT with 35 μg/mL of chloramphenicol and 100 μg/mL of ampicillin. 60 μL of the overnight cultures were inoculated to 6 mL of 2YT with antibiotics concentrations described previously and incubated in a shaker incubator at 37 °C until the optical density at 600 nm (OD_600_) reached 0.6. They are then induced with 0.2% arabinose. 3 mL of the culture was transferred into a culture tube containing 6 μL of 0.5 M BocK for 1 mM BocK expression of sfGFP. The tubes were collected after 6 h of expression at 37 °C. OD_600_ for each tube was measured in a Synergy neo2 plate reader. The cells were pelleted by centrifuging at 4000*g* for 10 min and then resuspended in a 500 μL fluorescence-detection buffer (20 mM tris, 100 mM NaCl, 100 μg/mL lysozyme, and 1 mM PMSF at pH 7.6). They were lysed via 3 freeze-and-thaw cycles in liquid nitrogen and a 37 °C dry bath separated by 20 s of vortexing. Inclusion bodies were removed by centrifugation at 16,500*g* for 30 min. 100 μL of supernatants were then aliquoted into 96-well plates (Grenier, 675076). The fluorescence was quantified by a neo2 plate reader with an excitation at 485 nm and an emission at 510 nm. The readout was normalized by dividing the OD_600_ values taken previously.

### Plasmid Construction for Ochre Suppression

pEVOL-MmPylS-MmPylT-UUA, pEVOL-CmaPylS-CmaPylT-UUA, and pBAD-sfGFP134TAA were constructed by QuikChange site-directed mutagenesis. The MmPylT mutation was introduced to pEVOL-MmPylRS/MmPylT by PCR with the following primers: MmPylT-UUA-f and MmPylT-UUA-r. The CmaPylT mutation was introduced into pEVOL-CmaPylRS/CmaPylT by PCR using the primers CmaPylT-UUA-f and CmaPylT-UUA-r. sfGFP134TAA was introduced to pBAD-sfGFP134TAG by the QuikChange site-directed mutagenesis using the primers sfGFP134TAA-f and sfGFP134TAA-r. The PCR products were processed by *Dpn*I and then used to directly transform Top10 *E. coli* competent cells. Colonies were collected and sequence-verified.

### Ochre Suppression Quantification

The plasmids with PylT-UUA were used to transform Top10 *E. coli* competent cells harboring pBAD-sfGFP134TAA. The fluorescence assay was performed as previously described.

### Construction of CmaPylT Variable Loop Mutations

C41A, C41CA, C41AU, and G43U mutations of CmaPylT were generated by the QuikChange site-directed mutagenesis on plasmids pEVOL-MmPylS/CmaPylT and pEVOL-CMaPylS/CmaPylT. A reverse primer CmaPylTvar-r was used with different forward primers, C41A-f, C41CA-f, C41AU-f, and G43U-f, to introduce mutations by PCR. The PCR products were treated with *Dpn*I and gel-extracted. They were then treated with T4 polynucleotide kinase at room temperature for 1 h and T4 DNA ligase at room temperature for 2 h. The product was directly used to transform Top10 *E. coli* competent cells and sequence-verified.

### Quantification of Variable Loop Mutations

pEVOL plasmids with CmaPylT C41A, C41CA, C41AU, or G43U are used to transform Top10 *E. coli* competent cells harboring pBAD-sfGFP134TAG. Single colonies were picked and inoculated to 5 mL of 2YT containing 35 μg/mL of chloramphenicol and 100 μg/mL of ampicillin for growing overnight cultures. The amber suppression and fluorescence assays were carried out as previously described to quantify the amber suppression for each mutant.

### Construction of pBK-CmaPylS and pY^+^-CmaPylT

The CmaPylS gene was amplified by PCR from pEVOL-CmaPylS/CmaPylT using the primer pairs pBK-Cma-f and pBK-Cma-r. The amplification product was digested with *Nde*I and *Pst*I and cloned to the pBK backbone, treated with the same restriction enzymes. The ligation product was then used to transform Top10 *E. coli* competent cells and sequence-verified.

The ProK promoter and CmaPylT were isolated from pEVOL-CmaPylS/CmaPylT by using the *Bgl*II restriction enzyme to digest the plasmid. It was then cloned to the pY^+^ backbone, which was digested by *Bgl*II and CIP at 37 °C for 10 h. The two fragments were ligated by T4 ligase at 16 °C overnight. The ligation product was then used to transform Top10 *E. coli* competent cells and sequence-verified. The verified plasmid was then split into two fragments by PCR using two primer pairs: pYPst-f and C41AU-f; pYPst-r and CmaPylTvar-r, to introduce the C41AU mutation. Both fragments were treated with *Pst*I followed by gel extraction. They were then mixed as a 1:1 ratio, phosphorylated with T4 polynucleotide kinase at 37 °C for 1 h, and then ligated with T4 DNA ligase at 16 °C overnight. The ligation product was then used to transform Top10 *E. coli* electrocompetent cells to maximize transformation and sequence-verified.

### Mutagenesis Protocol

The whole pBK-CmaPylS plasmid was randomized with error-prone PCR with primer pairs pBK-Cma-f and pBK-Cma-r. The PCR products were cleaned (Epoch, Gencatch mini-Prep 2160-050) and then used as templates for the second round of error-prone PCR. PCR products were verified by agarose gel electrophoresis. PCR products from both rounds were cleaned, combined, and then digested with *Pst*I and cloned into the pBK backbone (digested by *Nde*I and *Pst*I) using T4 DNA ligase overnight at 16 °C. The ligation product was then cleaned up with miniprep assay kits from Epoch and eluted into autoclaved Milli-Q water.

### Screening and Fluorescence Quantification

The ligation product was used to transform Top10 *E. coli* competent cells harboring pY^+^-CmaPylT-C41AU by electroporation. After recovery at 37 °C, multiple transformations were combined to form a CmaPylS library larger than 106. The library size was quantified by the serial dilution of the recovered culture in 2YT media without antibiotics, and 10 μL was spotted on agar plates containing 50 μg/mL of kanamycin and 10 μg/mL of tetracycline. It was then added to 1 L of 2YT media for amplifying overnight. The overnight culture was inoculated to 2YT with the desired antibiotics and 175 μg/mL of chloramphenicol and 0.1 mM BocK to perform live and death selection until it reached an OD_600_ at 0.5. The selected culture was diluted 100 times in 2YT, and 10 uL of the culture was plated onto agar plates (Kan, Tet, 0.2% arabinose, 1 mM IPTG) with various BocK concentrations (0.1, 0.5, 1 mM) to perform fluorescence selection. Fluorescent colonies were visualized under blue light and inoculated in 1 mL of 2YT containing kanamycin and tetracycline. After 6 h of incubation, OD_600_ of each colony was quantified with a plate reader. The cell concentration was normalized according to the most diluted colony by diluting with 2YT media. 10 uL of each culture was spotted onto LB agar plates (kan, tet, 0.2% arabinose, and 1 mM ITPG) with 0, 0.1, and 0.5 mM BocK for GFP_UV_ expression. After 18 h of incubation at 37 °C, the fluorescence was visualized under blue light. The fluorescent clones were identified and inoculated from 1 mL of culture to quantify fluorescence by plate reader. The fluorescence assay was carried out as previously described except that GFP_UV_ was expressed under 0.2% arabinose, 1 mM IPTG, and 1 mM BocK.

### The CmaPylS Gene Transferred from pBK to pEVOL

The genes of CmaPylS mutants were PCR-amplified using two primers, pBK 2pEVOL-f and pBK 2pEVOL-r. It is then restriction-digested with *Spe*I and *Sal*I and ligated to the pEVOL backbone containing CmaPylT-C41AU using T4 DNA ligase overnight at 16 °C. The ligated products were directly used to transform Top10 *E. coli* competent cells and sequence-verified.

### Fluorescence Assay from Screened Clones

The pEVOL-CmaPylS mutant plasmids were used to transform Top10 *E. coli* competent cells harboring pBAD-sfGFP134TAG. Single colonies from each transformation were inoculated to 5 mL of 2YT with ampicillin and chloramphenicol. The fluorescence assay was carried out as previously described.

### Third Round Selection with pEVOL

An error-prone PCR was performed on pEVOL-CmaPylS R2–7 with primer pairs pBK 2pEVOL-f and pBK 2pEVOL-r. It was cleaned using the Epoch mini-Prep kit and used as an error-prone PCR template. Both PCR products were combined and restriction-digested with *Spe*I and *Sal*I. It was then gel-extracted and ligated to the pEVOL backbone with T4 DNA ligase at 16 °C overnight. The ligated product was cleaned and eluted into autoclaved Milli-Q water and was then used to transform Top10 *E. coli* electrocompetent cells harboring pBAD-sfGFP134TAG. Multiple electroporation products were combined to afford a library larger than 106. The library size was quantified by a serial dilution of the recovered culture and spotted onto LB agar plates containing ampicillin and chloramphenicol. Colonies showing fluorescence were then picked.

### Fluorescence Quantification of Each Clone from the Third Round of Mutagenesis

The fluorescence quantification was carried out similarly to previous rounds of mutagenesis, except that sfGFP134BocK was expressed under 0.2% arabinose and 1 mM BocK.

### Construction of Double Incorporation Plasmids

pBAD-sfGFP1TAG134TAA was constructed by site-directed mutagenesis. 1TAG was introduced to pBAD-sfGFP134TAA by PCR using primers sfGFP1AlaTAG-f and sfGFP1AlaTAG-r. The PCR product was treated with *Dpn*I. It was then restriction-digested with *Nco*I at 37 °C for 10 h and ligated with T4 DNA ligase at 16 °C overnight. The ligation product was used to transform Top10 *E. coli* competent cells and sequence-verified. The verified plasmid was linearized by PCR using primers GFPdouble-f and GFPdouble-r to introduce *Xho*I and *Hin*dIII restriction cutting sites. Meanwhile, CmaPylS/CmaPylT-C41AU gene was PCR-amplified from pEVOL-CmaPylS/CmaPylT using the primer pairs C2G-f and C2G-r to introduce restriction cutting sites. The pBAD fragment was ligated with the CmaPylS/CmaPylT gene using T4 DNA ligase to afford the protein expression cassette with sfGFP and CmaPylS gene expression under control of the araBAD promoter. The ligation product was used to transform Top10 *E. coli* competent cells and sequence-verified. CmaPylS mutant genes were amplified from pEVOL-CmaPylS R3–9 and R3–14 with PCR primer pairs, pBK 2pEVOL-f and pBK 2pEVOL-r. They were restriction-digested by *Spe*I and *Sal*I and ligated to the pBAD-sfGFP1TAG134TAG-CmaPylT-C41AU backbone, which was treated as well with *Spe*I and *Sal*I. The ligation production pBAD-sfGFP1TAG134TAG-CmaPylS-CmaPylT-C41AU was then used to transform Top10 *E. coli* competent cells and sequence-verified.

### Double Incorporation Conditions

pBAD-sfGFP1TAG134TAA-CmaPylS-CmaPylT-C41AU with different CmaPylS clones were used to transform Top10 *E. coli* competent cells harboring pEVOL-AcKRS/MmPylT-UUA. Each colony was inoculated to 2YT containing ampicillin and chloramphenicol and incubated at 37 °C overnight. The overnight culture was inoculated to 2YT and incubated until it reached an OD_600_ of 0.6. Double incorporation was demonstrated by expressing sfGFP1BocK134AcK overnight by induction with 0.2% arabinose and various concentrations of AcK and BocK. The fluorescence was quantified as previously described.

### sfGFP1BocK134AcK Expression and Validation with ESI-MS

The Top10 *E. coli* cells harboring pEVOL-MmAcKS-MmPylT-UUA and pBAD-sfGFP1TAG134TAA-CmaPylS-CmaPylT-C41AU (the CmaPylS identity varied) were inoculated to 200 mL of 2YT with ampicillin and chloramphenicol and allowed to grow until OD_600_ reached 0.6. The sfGFP1BocK134AcK was expressed in the presence of 5 mM AcK and 10 mM BocK overnight. The cells were pelleted by centrifuging at 6000 rpm for 10 min. They were then resuspended in lysis buffer (20 mM tris, 100 mM NaCl, and 30 mM imidazole at pH 7.6) with 100 μg/mL of lysozyme and 1 mM of PMSF and lysed by sonication at a 65% amplitude for 5 min with 1 s on/4 s off. The cell lysates were separated by centrifuging at 14,000 rpm for 30 min. The supernatants were loaded onto Ni-NTA resins (Genscript, no. L00223). They were washed with 10 column volumes of lysis buffer and eluted with lysis buffer supplemented with 300 mM imidazole. The eluted sfGFP was concentrated to 1 mL with amicon (Millipore Sigma, UFC9050) and dialyzed to the lysis buffer with 1 mM EDTA. Protein purity was verified by SDS-PAGE.

The purified sfGFP was injected into a C4 HPLC column (AccucoreTM, 16526–102130) and eluted with a gradient of 0–40% acetonitrile with 0.2% formic acid. The eluent was injected into a QExactive Orbitrap mass spectrometer (ThermoFisher) for electrospray ionization mass spectrometry analysis. The collected mass spectra were then deconvoluted in Xcalibur with a 1 amu resolution. The peaks were identified with a threshold of 10%.

## Supporting Information Available

Experimental details for the construction of all plasmids, the construction of sequence-randomized CmaPylRS libraries, the plating and screening protocols, the expression of sfGFP incorporated with two different ncAAs, and the ESI-MS characterization of purified proteins (PDF)

## Abbreviations

ncAA: noncanonical amino acid
PylRS: pyrrolysyl-tRNA synthetase
CmaPylRS: *Ca. M. alvus* PylRS
MmPylRS: *M. mazei* PylRS
CmatRNA^Pyl^: *Ca. M. alvus* tRNA^Pyl^
MmtRNA^Pyl^: *M. mazei* tRNA^Pyl^
BocK: *N*^ε^-(*t*-butoxycarbonyl)-l-lysine
AcK: *N*^ε^-acetyl-l-lysine
GFP_UV_: ultraviolet light-excited green fluorescence protein
sfGFP: superfolder green fluorescent protein
