# Supplementary material for: Directed Evolution of Candidatus Methanomethylophilus alvus Pyrrolysyl-tRNA Synthetase for the Genetic Incorporation of Two Different Noncanonical Amino Acids in One Protein
Source: ACS Bio Med Chem Au. 2024 Aug 22;4(5):233–41. doi: 10.1021/acsbiomedchemau.4c00028 (PMC11487537; doi:10.1021/acsbiomedchemau.4c00028)
Supplement: Supplementary file 1 — bg4c00028_si_001.pdf [file bg4c00028_si_001.pdf]

Supplementary information for

Directed Evolution of *Candidatus Methanomethylophilus alvus* Pyrrolysyl-tRNA Synthetase for the Genetic Incorporation of Two Different Noncanonical Amino Acids in One Protein

Chia-Chuan D. Cho[a], Waye Michelle Leeuwon[b], and Wenshe Ray Liu[a,c,d,e,f,*]

[a]Texas A&M Drug Discovery Center and Department of Chemistry, College of Arts and Scienes, Texas A&M University, College Station, TX 77843, United States

[b]Cancer Prevention and Research Institute of Texas, Austin, TX 77843, United States

[c]Institute of Biosciences and Technology and Department of Translational Medical Sciences, School of Medicine, Texas A&M University, Houston, TX 77030, United States

[d]Department of Biochemistry and Biophysics, College of Agriculture and Life Sciences, Texas A&M University, College Station, TX 77843, United States

[e]Department of Cell Biology and Genetics, School of Medicine, Texas A&M University, College Station, TX 77843, United States

[f]Department of Pharmaceutical Sciences, Irma Lerma Rangel School of Pharmacy, Texas A&M University, College Station, TX 77843, United States

[*]Correspondence should be addressed to Wenshe Ray Liu: wsliu2007@tamu.edu

Supplementary Tables

1. Primer list

Primer name	product	sequence
CmaPylRS-for	pEVOL-CmaPylRS	GGAGGAATTACTAGTATGACGGTAAAGTACACAG
CmaPylRS-rev	pEVOL-CmaPylRS	CCCTGAGCCTCGAGAAAAAGCCTGCTCG
MmPylTUUA-f	MmPylTUUA	GAATGGACTTTAAATCCGTTCAG
MmPylTUUA-r	MmPylTUUA	CTGAACGGATTTAAAGTCCATTC
CmaPylTUUA-f	CmaPylTUUA	CAGCGGGTCTTTAAAACCTAGCCAG
CmaPylTUUA-t	CmaPylTUUA	CTGGCTAGGTTTTAAAGACCCGCTG
sfGFP134TAA-f	sfGFP134TAA	ATTTTAAAGAATAAGGCAACATTCT
sfGFP134TAA-r	sfGFP134TAA	AGAATGTTGCCTTATTCTTTAAAAT
CmaPylTvar-r	CmaPylT variable loop mutation	GCTAGGTTTTAGAGACCC
C41A -f	pEVOL-CmaPylRS/CmaPylTC41A	AAGCGGGGTTCGAC
C41CA-f	pEVOL-CmaPylRS/CmaPylTC41CA	CAAGCGGGGTTCGAC
C41AU-f	pEVOL-CmaPylRS/CmaPylTC41AU	ATAGCGGGGTTCGAC

G43U-f	pEVOL-CmaPylRS/CmaPylTG43U	CATCGGGGTTCGAC
pBK-Cma-f	pBK-CmaPylRS	GAGGAATCCCATATGACGGTAAAGTAC
pBK-Cma-r	pBK-CmaPylRS	GAAACTGCAGTCAGTTGATCTTGGCC
pYPst-f	pY+CmaPylTC41AU	ACGGTCACAGCCTGCAGCAAAAAACCCCTCAAG
pYPst-r	pY+CmaPylTC41AU	CTTGAGGGGTTTTTTGCTGCAGGCTGTGACCGT
pBK2pEVOL-f	tranfer CmaPylRS to pEVOL	AATTACTAGTATGACGGTAAAGTACACAGATGCTCAGATCC
pBK2pEVOL-r	tranfer CmaPylRS to pEVOL	CTCAGTCGACTCAGTTGATCTTGGCCCC
sfGFP1AlaTAG-f	pBAD-sfGFP1TAG134TAA	GCGTAGGTTAGCAAAGGTGAAGAACTGTTTAC
sfGFP1AlaTAG-r	pBAD-sfGFP1TAG134TAA	CATGGTTAATTCCTCCTGTTAGCCCAA
GFPdouble-r	double incorporation plasmid	TTCGAAGCTTGGCTGTTTTGGCG
GFPdouble-r	double incorporation plasmid	CAGATCTCGAGCTTCAATGGTGATGATGAT
C2G-f	double incorporation plasmid	ACTTCTCGAGAGGAGGAATTACTAGTATGACG
C2G-r	double incorporation plasmid	AATTAAGCTTCAGGCTTTTCGAATTTGG

Supplementary Figures

(A)

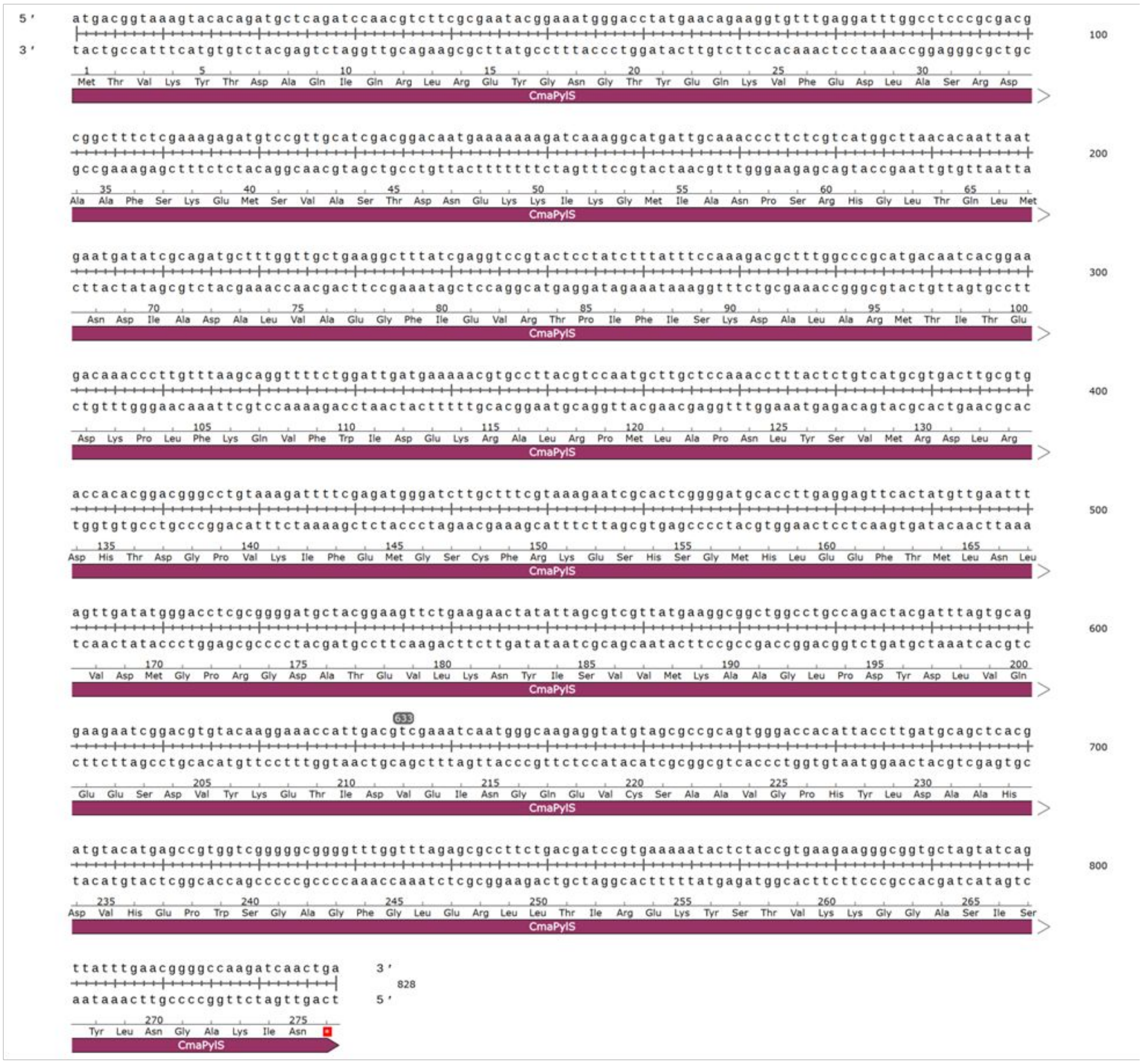

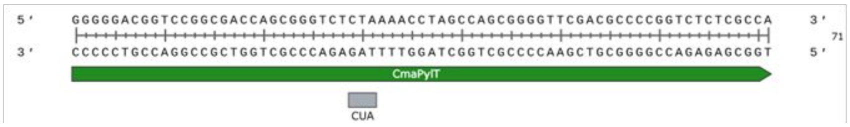

CmaPylRS DNA sequence

ATGACGGTAAAGTACACAGATGCTCAGATCCAACGTCTTCGCGAATACGGAAATGGGACC
TATGAACAGAAGGTGTTTGAGGATTTGGCCTCCCGCGACGCGGCTTTCTCGAAAGAGATG
TCCGTTGCATCGACGGACAATGAAAAAAAGATCAAAGGCATGATTGCAAACCCTTCTCGT
CATGGCTTAACACAATTAATGAATGATATCGCAGATGCTTTGGTTGCTGAAGGCTTTATCG
AGGTCCGTACTCCTATCTTTATTTCCAAAGACGCTTTGGCCCGCATGACAATCACGGAAG
ACAAACCCTTGTTTAAGCAGGTTTTCTGGATTGATGAAAAACGTGCCTTACGTCCAATGCT
TGCTCCAAACCTTTACTCTGTCATGCGTGACTTGCGTGACCACACGGACGGGCCTGTAAA
GATtTTCGAGATGGGATCTTGCTTTCGTAAAGAATCGCACTCGGGGATGCACCTTGAGGA
GTTCACTATGTTGAATTTAGTTGATATGGGACCTCGCGGGGATGCTACGGAAGTTCTGAA
GAACTATATTAGCGTCGTTATGAAGGCGGCTGGCCTGCCAGACTACGATTTAGTGCAGGA
AGAATCGGACGTGTACAAGGAAACCATTGACGTCGAAATCAATGGGCAAGAGGTATGTAG
CGCCGCAGTGGGACCACATTACCTTGATGCAGCTCACGATGTACATGAGCCGTGGTCGG
GGGCGGGGTTTGGTTTAGAGCGCCTTCTGACGATCCGTGAAAAATACTCTACCGTGAAGA
AGGGCGGTGCTAGTATCAGTTATTTGAACGGGGCCAAGATCAACTGA

CmaPylT DNA sequence

GGGGGACGGTCCGGCGACCAGCGGGTCTCTAAAACCTAGCCAGCGGGGTTCGACGCCC
CGGTCTCTCGCCA

MmPylRS DNA sequence

ATGGATAAGAAACCGCTGAATACTCTGATTTCTGCAACTGGTCTGTGGATGAGCCGTACC
GGCACCATCCACAAGATCAAACACCACGAGGTTTCCCGTAGCAAAATCTACATCGAAATG
GCGTGCGGTGACCACCTGGTGGTAAACAACTCCCGTTCTTCTCGTACTGCACGTGCTCTG
CGCCACCACAAGTACCGTAAGACCTGCAAGCGCTGTCGCGTGTCTGATGAAGACCTGAA
CAAATTCCTGACTAAAGCGAACGAAGATCAGACTTCTGTGAAGGTGAAAGTTGTTTCTGC
CCCAACCCGCACCAAGAAAGCGATGCCGAAGTCCGTTGCACGCGCTCCGAAACCGCTGG
AGAACACCGAAGCCGCACAGGCCCAGCCGTCTGGTTCTAAGTTTTCTCCGGCAATCCCG
GTTTCTACTCAGGAGTCTGTGTCTGTGCCAGCTTCTGTTAGCACTTCTATTTCCTCTATCA
GCACTGGTGCGACTGCGTCCGCTCTGGTAAAAGGTAACACTAACCCGATCACCAGCATG
TCTGCTCCGGTTCAGGCTTCTGCACCGGCACTGACTAAAAGCCAGACTGACCGTCTGGA
GGTTCTGCTGAACCCGAAAGATGAAATCAGCCTGAACTCTGGCAAACCGTTCCGTGAACT
GGAATCCGAACTGCTGTCTCGTCGTAAGAAAGACCTGCAACAAATCTATGCTGAAGAGCG
TGAAAACTACCTGGGTAAACTGGAACGTGAAATCACCCGTTTCTTTGTGGACCGTGGTTT
CCTGGAAATCAAGTCTCCGATCCTGATCCCGCTGGAATACATCGAGCGCATGGGTATTGA
TAACGACACCGAACTGTCCAAGCAGATTTTCCGTGTGGACAAGAACTTCTGCCTGCGTCC
GATGCTGGCACCGAACCTGTACAATTACCTGCGTAAACTGGATCGTGCACTGCCGGACC
CGATCAAAATCTTTGAAATCGGTCCATGCTATCGTAAGGAGAGCGACGGTAAAGAACACC
TGGAAGAGTTCACTATGCTGAACTTTTGTCAGATGGGTTCTGGCTGCACCCGTGAAAATC
TGGAATCTATCATCACCGACTTCCTGAACCACCTGGGCATTGACTTCAAAATCGTTGGTGA
TTCCTGCATGGTTTACGGTGACACTCTGGACGTTATGCATGGTGATCTGGAACTGAGCAG
CGCTGTTGTGGGTCCGATTCCGCTGGATCGTGAATGGGGTATCGATAAACCGTGGATTG
GTGCTGGCTTCGGTCTGGAACGTCTGCTGAAAGTTAAGCACGACTTTAAGAACATCAAAC
GTGCTGCGCGTTCCGAGTCCTATTACAACGGCATTAGCACTAACCTGTAA

MmPylT

GGAAACCTGATCATGTAGATCGAATGGACTCTAAATCCGTTCAGCCGGGTTAGATTCCCG
GGGTTTCCGCCA

(B)

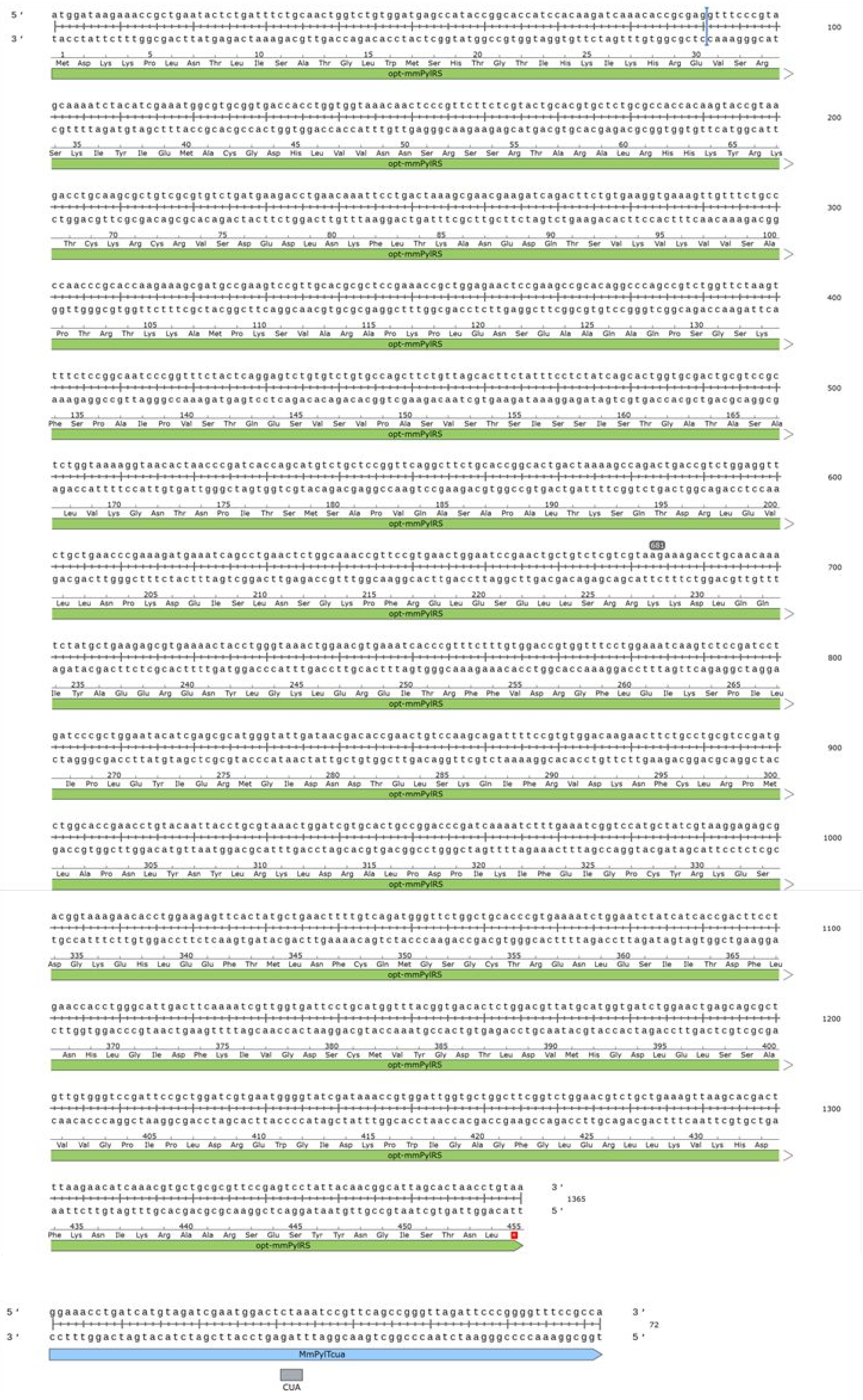

(C)

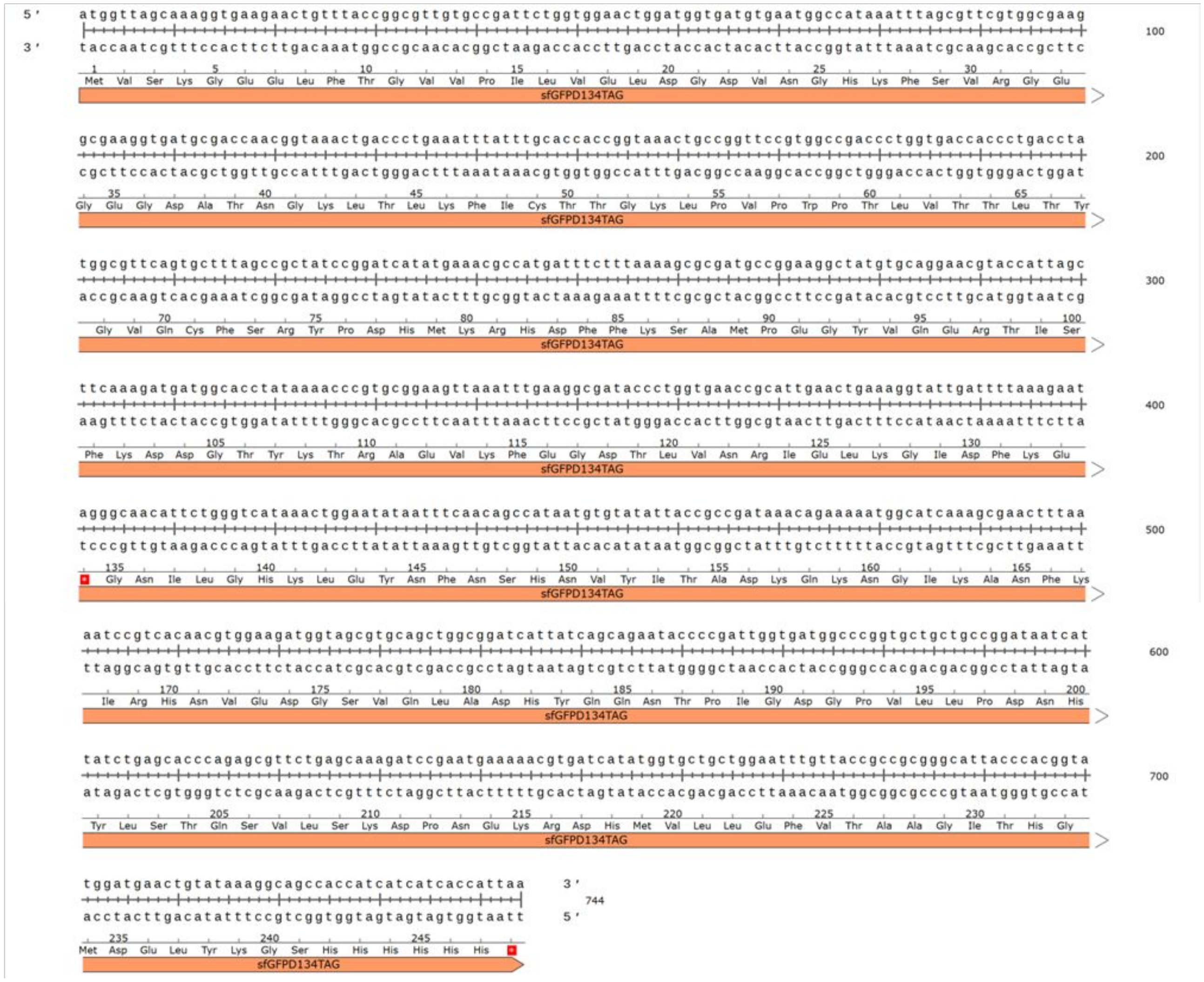

sfGFP134TAG

atggttagcaaaggtgaagaactgtttaccggcgttgtgccgattctggtggaactggatggtgatgtgaatggccataaatttagcgtt
cgtggcgaaggcgaaggtgatgcgaccaacggtaaactgaccctgaaatttatttgcaccaccggtaaactgccggttccgtggcc
gaccctggtgaccaccctgacctatggcgttcagtgctttagccgctatccggatcatatgaaacgccatgatttctttaaaagcgcgat
gccggaaggctatgtgcaggaacgtaccattagcttcaaagatgatggcacctataaaacccgtgcggaagttaaatttgaaggcg
ataccctggtgaaccgcattgaactgaaaggtattgattttaaagaatagggcaacattctgggtcataaactggaatataatttcaac
agccataatgtgtatattaccgccgataaacagaaaaatggcatcaaagcgaactttaaaatccgtcacaacgtggaagatggtag
cgtgcagctggcggatcattatcagcagaataccccgattggtgatggcccggtgctgctgccggataatcattatctgagcaccca
gagcgttctgagcaaagatccgaatgaaaaacgtgatcatatggtgctgctggaatttgttaccgccgcgggcattacccacggtat
ggatgaactgtataaaggcagccaccatcatcatcaccattaa

Figure S1. DNA and protein sequences for (**A**) CmaPylS and CmaPylT; (**B**) MmPylS and MmPylT; (**C**) sfGFP with the D134 coding position mutated to amber codon.

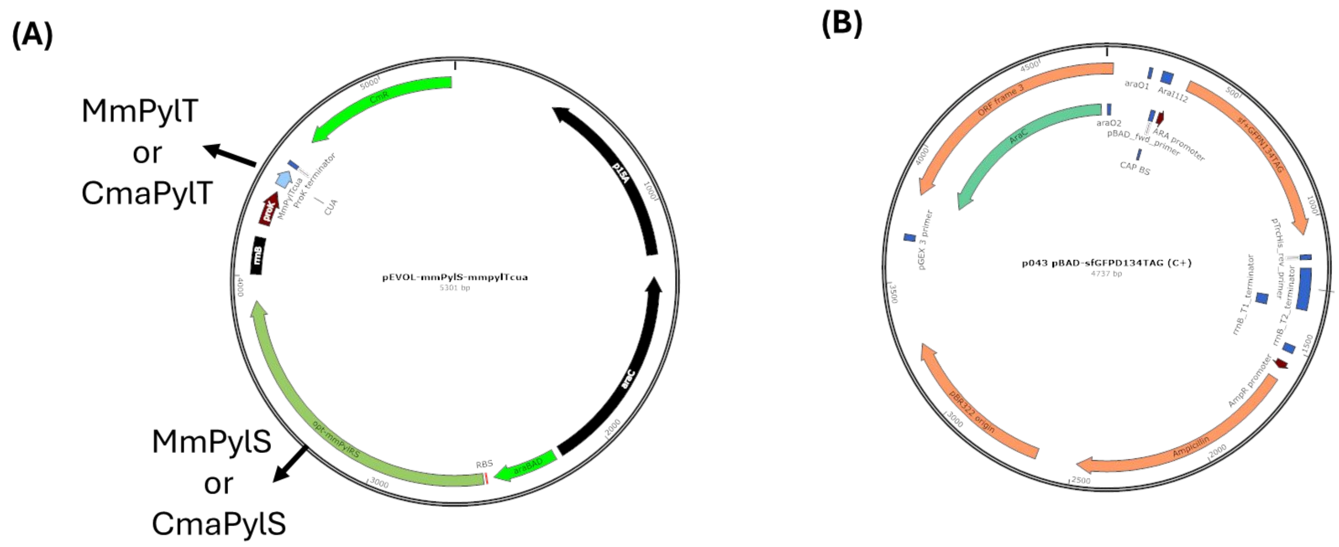

pEVOL-CmaPylS-CmaPylT plasmid map

tcctgaaaatctcgataactcaaaaaatacgcccggtagtgatcttatttcattatggtgaaagttggaacctcttacgtgccgatcaac
gtctcattttcgccaaaagttggcccagggcttcccggtatcaacagggacaccaggatttatttattctgcgaagtgatcttccgtcac
aggtatttattcggcgcaaagtgcgtcgggtgatgctgccaacttactgatttagtgtatgatggtgtttttgaggtgctccagtggcttctgt
ttctatcagctgtccctcctgttcagctactgacggggtggtgcgtaacggcaaaagcaccgccggacatcagcgctagcggagtgt
atactggcttactatgttggcactgatgagggtgtcagtgaagtgcttcatgtggcaggagaaaaaaggctgcaccggtgcgtcagc
agaatatgtgatacaggatatattccgcttcctcgctcactgactcgctacgctcggtcgttcgactgcggcgagcggaaatggcttac
gaacggggcggagatttcctggaagatgccaggaagatacttaacagggaagtgagagggccgcggcaaagccgtttttccata
ggctccgcccccctgacaagcatcacgaaatctgacgctcaaatcagtggtggcgaaacccgacaggactataaagataccagg
cgtttccccctggcggctccctcgtgcgctctcctgttcctgcctttcggtttaccggtgtcattccgctgttatggccgcgtttgtctcattcca
cgcctgacactcagttccgggtaggcagttcgctccaagctggactgtatgcacgaaccccccgttcagtccgaccgctgcgccttat
ccggtaactatcgtcttgagtccaacccggaaagacatgcaaaagcaccactggcagcagccactggtaattgatttagaggagtt
agtcttgaagtcatgcgccggttaaggctaaactgaaaggacaagttttggtgactgcgctcctccaagccagttacctcggttcaaa
gagttggtagctcagagaaccttcgaaaaaccgccctgcaaggcggttttttcgttttcagagcaagagattacgcgcagaccaaaa
cgatctcaagaagatcatcttattaatcagataaaatatttctagatttcagtgcaatttatctcttcaaatgtagcacctgaagtcagccc
catacgatataagttgtaattctcatgtttgacagcttatcatcgataagcttggtacccaattatgacaacttgacggctacatcattcact
ttttcttcacaaccggcacggaactcgctcgggctggccccggtgcattttttaaatacccgcgagaaatagagttgatcgtcaaaacc
aacattgcgaccgacggtggcgataggcatccgggtggtgctcaaaagcagcttcgcctggctgatacgttggtcctcgcgccagct
taagacgctaatccctaactgctggcggaaaagatgtgacagacgcgacggcgacaagcaaacatgctgtgcgacgctggcgat
atcaaaattgctgtctgccaggtgatcgctgatgtactgacaagcctcgcgtacccgattatccatcggtggatggagcgactcgttaa
tcgcttccatgcgccgcagtaacaattgctcaagcagatttatcgccagcagctccgaatagcgcccttccccttgcccggcgttaatg
atttgcccaaacaggtcgctgaaatgcggctggtgcgcttcatccgggcgaaagaaccccgtattggcaaatattgacggccagtta
agccattcatgccagtaggcgcgcggacgaaagtaaacccactggtgataccattcgcgagcctccggatgacgaccgtagtgat
gaatctctcctggcgggaacagcaaaatatcactcggtcggcaaacaaattctcgtccctgatttttcaccaccccctgaccgcgaat
ggtgagattgagaatataacctttcattcccagcggtcggtcgataaaaaaatcgagataaccgttggcctcaatcggcgttaaaccc
gccaccagatgggcattaaacgagtatcccggcagcaggggatcattttgcgcttcagccatacttttcatactcccgccattcagag
aagaaaccaattgtccatattgcatcagacattgccgtcactgcgtcttttactggctcttctcgctaaccaaaccggtaaccccgcttat
taaaagcattctgtaacaaagcgggaccaaagccatgacaaaaacgcgtaacaaaagtgtctataatcacggcagaaaagtcc

acattgattatttgcacggcgtcacactttgctatgccatagcatttttatccataagattagcggatcctacctgacgctttttatcgcaact
ctctactgtttctccatacccgtttttttgggctaacaggaggaattactagtATGACGGTAAAGTACACAGATGCTCA
GATCCAACGTCTTCGCGAATACGGAAATGGGACCTATGAACAGAAGGTGTTTGAGGATTT
GGCCTCCCGCGACGCGGCTTTCTCGAAAGAGATGTCCGTTGCATCGACGGACAATGAAA
AAAAGATCAAAGGCATGATTGCAAACCCTTCTCGTCATGGCTTAACACAATTAATGAATGA
TATCGCAGATGCTTTGGTTGCTGAAGGCTTTATCGAGGTCCGTACTCCTATCTTTATTTCC
AAAGACGCTTTGGCCCGCATGACAATCACGGAAGACAAACCCTTGTTTAAGCAGGTTTTC
TGGATTGATGAAAAACGTGCCTTACGTCCAATGCTTGCTCCAAACCTTTACTCTGTCATGC
GTGACTTGCGTGACCACACGGACGGGCCTGTAAAGATtTTCGAGATGGGATCTTGCTTTC
GTAAAGAATCGCACTCGGGGATGCACCTTGAGGAGTTCACTATGTTGAATTTAGTTGATAT
GGGACCTCGCGGGGATGCTACGGAAGTTCTGAAGAACTATATTAGCGTCGTTATGAAGG
CGGCTGGCCTGCCAGACTACGATTTAGTGCAGGAAGAATCGGACGTGTACAAGGAAACC
ATTGACGTCGAAATCAATGGGCAAGAGGTATGTAGCGCCGCAGTGGGACCACATTACCTT
GATGCAGCTCACGATGTACATGAGCCGTGGTCGGGGGCGGGGTTTGGTTTAGAGCGCCT
TCTGACGATCCGTGAAAAATACTCTACCGTGAAGAAGGGCGGTGCTAGTATCAGTTATTT
GAACGGGGCCAAGATCAACTGAgtcgactgagtttaaacggtctccagcttggctgttttggcggatgagagaagattt
tcagcctgatacagattaaatcagaacgcagaagcggtctgataaaacagaatttgcctggcggcagtagcgcggtggtcccacct
gaccccatgccgaactcagaagtgaaacgccgtagcgccgatggtagtgtggggtctccccatgcgagagtagggaactgccag
gcatcaaataaaacgaaaggctcagtcgaaagactgggcctttgtttgtgagctcacatgtgagcaccggtttattgactaccggaag
cagtgtgaccgtgtgcttctcaaatgcctgaggccagtttgctcaggctctccccgtggaggtaataattgacgatatgatcagtgcac
ggctaactaagcggcctgctgactttctcgccgatcaaaaggcattttgctattaagggattgacgagggcgtatctgcgcagtaaga
tgcgccccgcattGGGGGACGGTCCGGCGACCAGCGGGTCTCTAAAACCTAGCCAGCGGGGT
TCGACGCCCCGGTCTCTCGCCAaattcgaaaagcctgctcaacgagcaggctttttctcgaggctcagggtcgaatt
tgctttcgaatttctgccattcatccgcttattatcacttattcaggcgtagcaccaggcgtttaagggcaccaataactgccttaaaaaa
attacgccccgccctgccactcatcgcagtactgttgtaattcattaagcattctgccgacatggaagccatcacagacggcatgatg
aacctgaatcgccagcggcatcagcaccttgtcgccttgcgtataatatttgcccatggtgaaaacgggggcgaagaagttgtccat
attggccacgtttaaatcaaaactggtgaaactcacccagggattggctgagacgaaaaacatattctcaataaaccctttagggaa
ataggccaggttttcaccgtaacacgccacatcttgcgaatatatgtgtagaaactgccggaaatcgtcgtggtattcactccagagc
gatgaaaacgtttcagtttgctcatggaaaacggtgtaacaagggtgaacactatcccatatcaccagctcaccgtctttcattgccat
acggaattccggatgagcattcatcaggcgggcaagaatgtgaataaaggccggataaaacttgtgcttatttttctttacggtctttaa
aaaggccgtaatatccagctgaacggtctggttataggtacattgagcaactgactgaaatgcctcaaaatgttctttacgatgccatt
gggatatatcaacggtggtatatccagtgatttttttctccattttagcttccttagc

The highlighted sequence are PylRS and its corresponded PylT.

pBAD-sfGFP plasmid map

AAGAAACCAATTGTCCATATTGCATCAGACATTGCCGTCACTGCGTCTTTTACTGGCTCTT
CTCGCTAACCAAACCGGTAACCCCGCTTATTAAAAGCATTCTGTAACAAAGCGGGACCAA
AGCCATGACAAAAACGCGTAACAAAAGTGTCTATAATCACGGCAGAAAAGTCCACATTGA
TTATTTGCACGGCGTCACACTTTGCTATGCCATAGCATTTTTATCCATAAGATTAGCGGAT
CCTACCTGACGCTTTTTATCGCAACTCTCTACTGTTTCTCCATACCCGTTTTTTTGGGCTAAC
AGGAGGAATTAACCATGGTTAGCAAAGGTGAAGAACTGTTTACCGGCGTTGTGCCGATTC
TGGTGGAACTGGATGGTGATGTGAATGGCCATAAATTTAGCGTTCGTGGCGAAGGCGAA

GGTGATGCGACCAACGGTAAACTGACCCTGAAATTTATTTGCACCACCGGTAAACTGCCG
GTTCCGTGGCCGACCCTGGTGACCACCCTGACCTATGGCGTTCAGTGCTTTAGCCGCTAT
CCGGATCATATGAAACGCCATGATTTCTTTAAAAGCGCGATGCCGGAAGGCTATGTGCAG
GAACGTACCATTAGCTTCAAAGATGATGGCACCTATAAAACCCGTGCGGAAGTTAAATTTG
AAGGCGATACCCTGGTGAACCGCATTGAACTGAAAGGTATTGATTTTAAAGAATAGGGCA
ACATTCTGGGTCATAAACTGGAATATAATTTCAACAGCCATAATGTGTATATTACCGCCGA
TAAACAGAAAAATGGCATCAAAGCGAACTTTAAAATCCGTCACAACGTGGAAGATGGTAG
CGTGCAGCTGGCGGATCATTATCAGCAGAATACCCCGATTGGTGATGGCCCGGTGCTGC
TGCCGGATAATCATTATCTGAGCACCCAGAGCGTTCTGAGCAAAGATCCGAATGAAAAAC
GTGATCATATGGTGCTGCTGGAATTTGTTACCGCCGCGGGCATTACCCACGGTATGGATG
AACTGTATAAAGGCAGCCACCATCATCATCACCATTAAAGCTCGAGATCTGCAGCTGGTA
CCATATGGGAATTCGAAGCTTGGCTGTTTTGGCGGATGAGAGAAGATTTTCAGCCTGATA
CAGATTAAATCAGAACGCAGAAGCGGTCTGATAAAACAGAATTTGCCTGGCGGCAGTAGC
GCGGTGGTCCCACCTGACCCCATGCCGAACTCAGAAGTGAAACGCCGTAGCGCCGATG
GTAGTGTGGGGTCTCCCCATGCGAGAGTAGGGAACTGCCAGGCATCAAATAAAACGAAA
GGCTCAGTCGAAAGACTGGGCCTTTCGTTTTATCTGTTGTTTGTCGGTGAACGCTCTCCT
GAGTAGGACAAATCCGCCGGGAGCGGATTTGAACGTTGCGAAGCAACGGCCCGGAGGG
TGGCGGGCAGGACGCCCGCCATAAACTGCCAGGCATCAAATTAAGCAGAAGGCCATCCT
GACGGATGGCCTTTTTGCGTTTCTACAAACTCTTTTGTTTATTTTTCTAAATACATTCAAATA
TGTATCCGCTCATGAGACAATAACCCTGATAAATGCTTCAATAATATTGAAAAAGGAAGAG
TATGAGTATTCAACATTTCCGTGTCGCCCTTATTCCCTTTTTTGCGGCATTTTGCCTTCCTG
TTTTTGCTCACCCAGAAACGCTGGTGAAAGTAAAAGATGCTGAAGATCAGTTGGGTGCAC
GAGTGGGTTACATCGAACTGGATCTCAACAGCGGTAAGATCCTTGAGAGTTTTCGCCCCG
AAGAACGTTTTCCAATGATGAGCACTTTTAAAGTTCTGCTATGTGGCGCGGTATTATCCCG
TGTTGACGCCGGGCAAGAGCAACTCGGTCGCCGCATACACTATTCTCAGAATGACTTGGT
TGAGTACTCACCAGTCACAGAAAAGCATCTTACGGATGGCATGACAGTAAGAGAATTATG
CAGTGCTGCCATAACCATGAGTGATAACACTGCGGCCAACTTACTTCTGACAACGATCGG
AGGACCGAAGGAGCTAACCGCTTTTTTGCACAACATGGGGGATCATGTAACTCGCCTTGA
TCGTTGGGAACCGGAGCTGAATGAAGCCATACCAAACGACGAGCGTGACACCACGATGC
CTGTAGCAATGGCAACAACGTTGCGCAAACTATTAACTGGCGAACTACTTACTCTAGCTTC
CCGGCAACAATTAATAGACTGGATGGAGGCGGATAAAGTTGCAGGACCACTTCTGCGCT
CGGCCCTTCCGGCTGGCTGGTTTATTGCTGATAAATCTGGAGCCGGTGAGCGTGGGTCT
CGCGGTATCATTGCAGCACTGGGGCCAGATGGTAAGCCCTCCCGTATCGTAGTTATCTAC
ACGACGGGGAGTCAGGCAACTATGGATGAACGAAATAGACAGATCGCTGAGATAGGTGC
CTCACTGATTAAGCATTGGTAACTGTCAGACCAAGTTTACTCATATATACTTTAGATTGATT
TAAAACTTCATTTTTAATTTAAAAGGATCTAGGTGAAGATCCTTTTTGATAATCTCATGACC
AAAATCCCTTAACGTGAGTTTTCGTTCCACTGAGCGTCAGACCCCGTAGAAAAGATCAAA
GGATCTTCTTGAGATCCTTTTTTTCTGCGCGTAATCTGCTGCTTGCAAACAAAAAAACCAC
CGCTACCAGCGGTGGTTTGTTTGCCGGATCAAGAGCTACCAACTCTTTTTCCGAAGGTAA
CTGGCTTCAGCAGAGCGCAGATACCAAATACTGTCCTTCTAGTGTAGCCGTAGTTAGGCC
ACCACTTCAAGAACTCTGTAGCACCGCCTACATACCTCGCTCTGCTAATCCTGTTACCAGT
GGCTGCTGCCAGTGGCGATAAGTCGTGTCTTACCGGGTTGGACTCAAGACGATAGTTAC

CGGATAAGGCGCAGCGGTCGGGCTGAACGGGGGGTTCGTGCACACAGCCCAGCTTGGA
GCGAACGACCTACACCGAACTGAGATACCTACAGCGTGAGCTATGAGAAAGCGCCACGC
TTCCCGAAGGGAGAAAGGCGGACAGGTATCCGGTAAGCGGCAGGGTCGGAACAGGAGA
GCGCACGAGGGAGCTTCCAGGGGGAAACGCCTGGTATCTTTATAGTCCTGTCGGGTTTC
GCCACCTCTGACTTGAGCGTCGATTTTTGTGATGCTCGTCAGGGGGGCGGAGCCTATGG
AAAAACGCCAGCAACGCGGCCTTTTTACGGTTCCTGGCCTTTTGCTGGCCTTTTGCTCAC
ATGTTCTTTCCTGCGTTATCCCCTGATTCTGTGGATAACCGTATTACCGCCTTTGAGTGAG
CTGATACCGCTCGCCGCAGCCGAACGACCGAGCGCAGCGAGTCAGTGAGCGAGGAAGC
GGAAGAGCGCCTGATGCGGTATTTTCTCCTTACGCATCTGTGCGGTATTTCACACCGCAT
ATGGTGCACTCTCAGTACAATCTGCTCTGATGCCGCATAGTTAAGCCAGTATACACTCCG
CTATCGCTACGTGACTGGGTCATGGCTGCGCCCCGACACCCGCCAACACCCGCTGACGC
GCCCTGACGGGCTTGTCTGCTCCCGGCATCCGCTTACAGACAAGCTGTGACCGTCTCCG
GGAGCTGCATGTGTCAGAGGTTTTCACCGTCATCACCGAAACGCGCGAGGCAGCAGATC
AATTCGCGCGCGAAGGCGAAGCGGCATGCATAATGTGCCTGTCAAATGGACGAAGCAGG
GATTCTGCAAACCCTATGCTACTCCGTCAAGCCGTCAATTGTCTGATTCGTTACCAATTAT
GACAACTTGACGGCTACATCATTCACTTTTTCTTCACAACCGGCACGGAACTCGCTCGGG
CTGGCCCCGGTGCATTTTTTAAATACCCGCGAGAAATAGAGTTGATCGTCAAAACCAACA
TTGCGACCGACGGTGGCGATAGGCATCCGGGTGGTGCTCAAAAGCAGCTTCGCCTGGCT
GATACGTTGGTCCTCGCGCCAGCTTAAGACGCTAATCCCTAACTGCTGGCGGAAAAGATG
TGACAGACGCGACGGCGACAAGCAAACATGCTGTGCGACGCTGGCGATATCAAAATTGC
TGTCTGCCAGGTGATCGCTGATGTACTGACAAGCCTCGCGTACCCGATTATCCATCGGTG
GATGGAGCGACTCGTTAATCGCTTCCATGCGCCGCAGTAACAATTGCTCAAGCAGATTTA
TCGCCAGCAGCTCCGAATAGCGCCCTTCCCCTTGCCCGGCGTTAATGATTTGCCCAAACA
GGTCGCTGAAATGCGGCTGGTGCGCTTCATCCGGGCGAAAGAACCCCGTATTGGCAAAT
ATTGACGGCCAGTTAAGCCATTCATGCCAGTAGGCGCGCGGACGAAAGTAAACCCACTG
GTGATACCATTCGCGAGCCTCCGGATGACGACCGTAGTGATGAATCTCTCCTGGCGGGA
ACAGCAAAATATCACCCGGTCGGCAAACAAATTCTCGTCCCTGATTTTTCACCACCCCCT
GACCGCGAATGGTGAGATTGAGAATATAACCTTTCATTCCCAGCGGTCGGTCGATAAAAA
AATCGAGATAACCGTTGGCCTCAATCGGCGTTAAACCCGCCACCAGATGGGCATTAAACG
AGTATCCCGGCAGCAGGGGATCATTTTGCGCTTCAGCCATACTTTTCATACTCCCGCCAT
TCAGAG

Figure S2. Plasmid maps for (**A**) the pEVOL backbone with either or MmPylS/CmaPylS and MmPylT/CmaPylT; (**B**) pBAD-sfGFP.

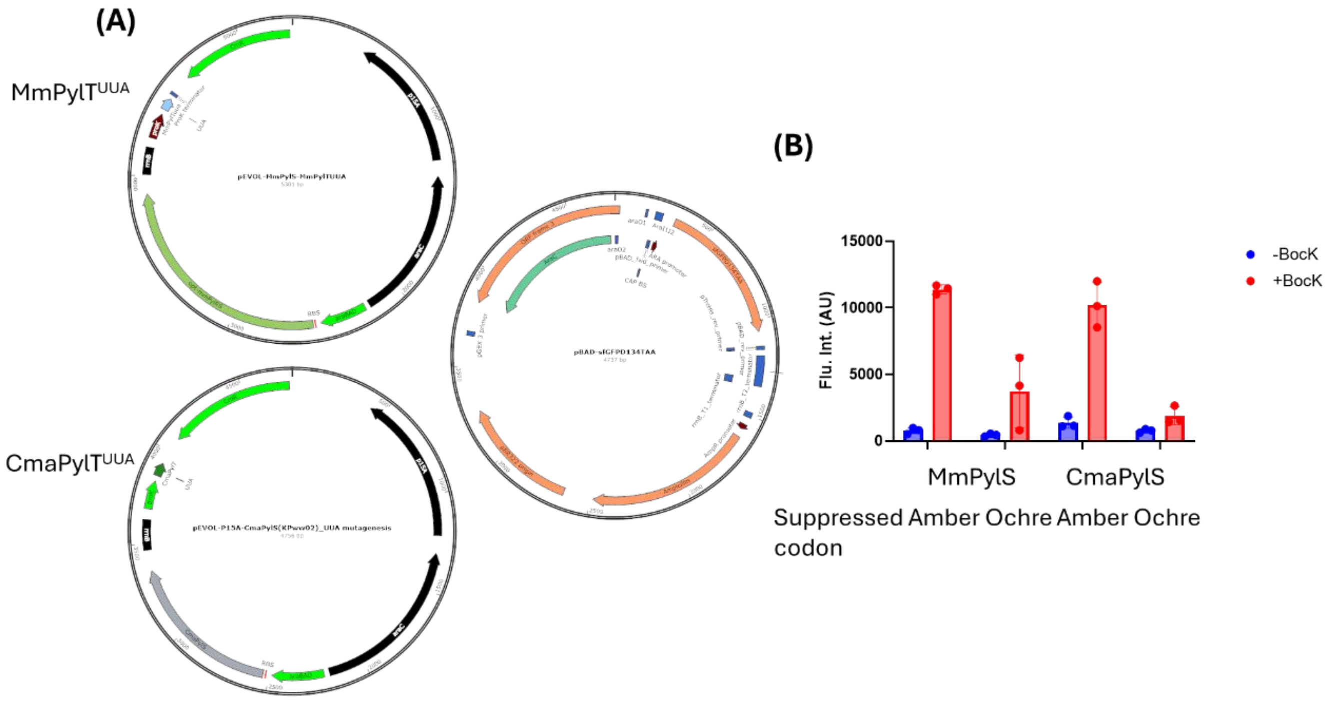

Figure S3. (**A**) plasmids for ochre suppression. (**B**) Comparison of ochre suppression by MmPylS and CmaPylS.

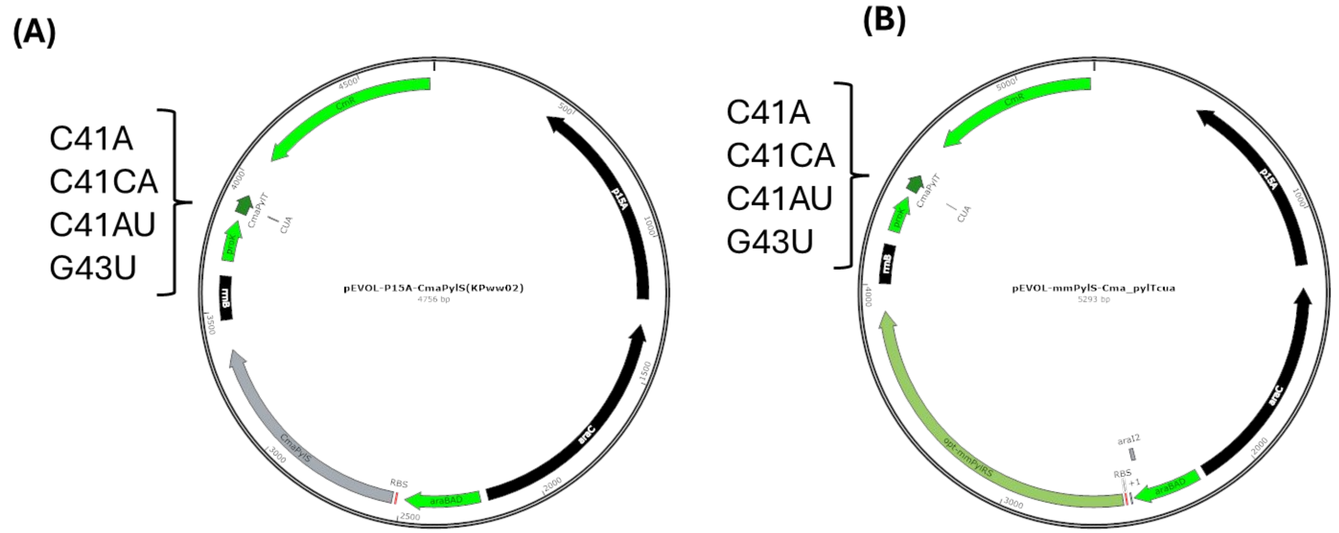

Figure S4. Plasmids for CmaPylT variable loop mutations: (**A**) pEVOL-CmaPylS-CmaPylT (**B**) pEVOL-MmPylS-MmPylT.

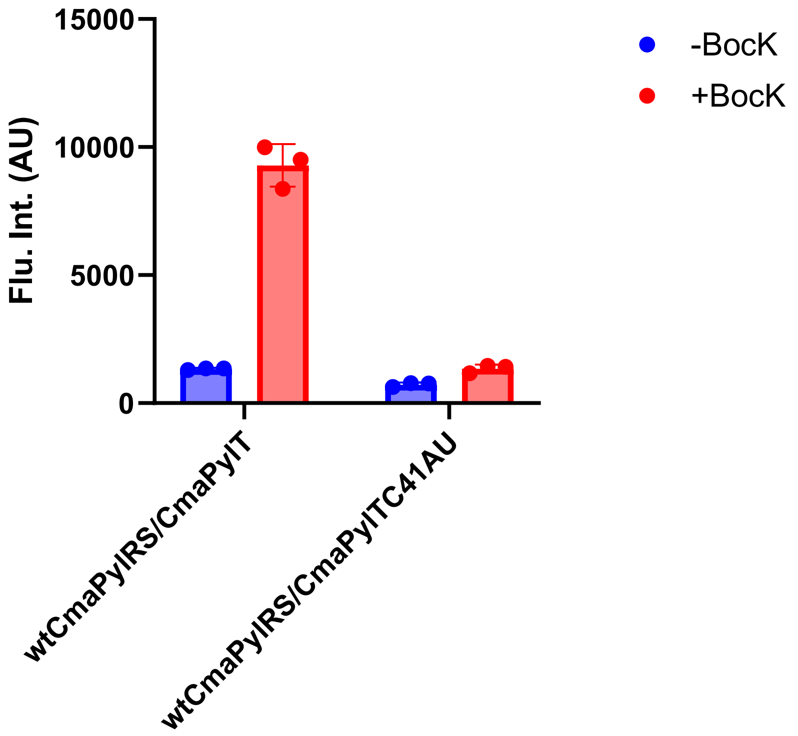

Figure S5. Comparison of amber suppression by CmaPylS/CmaPylT and CmaPylS/CmaPylT-C41AU.

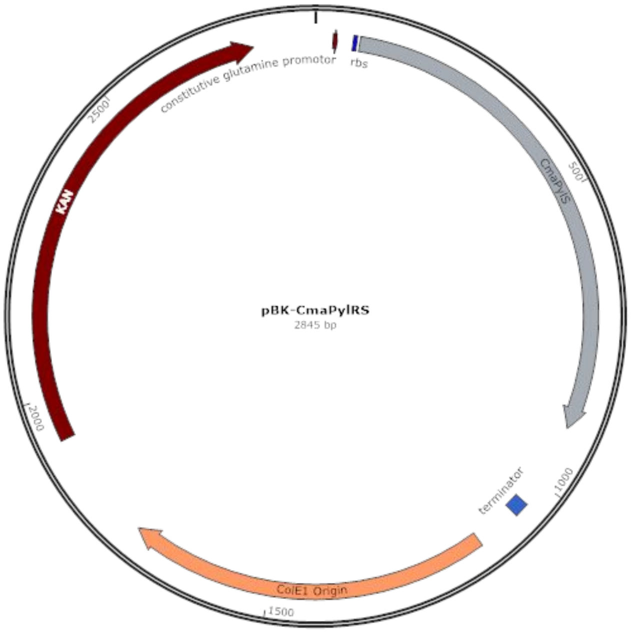

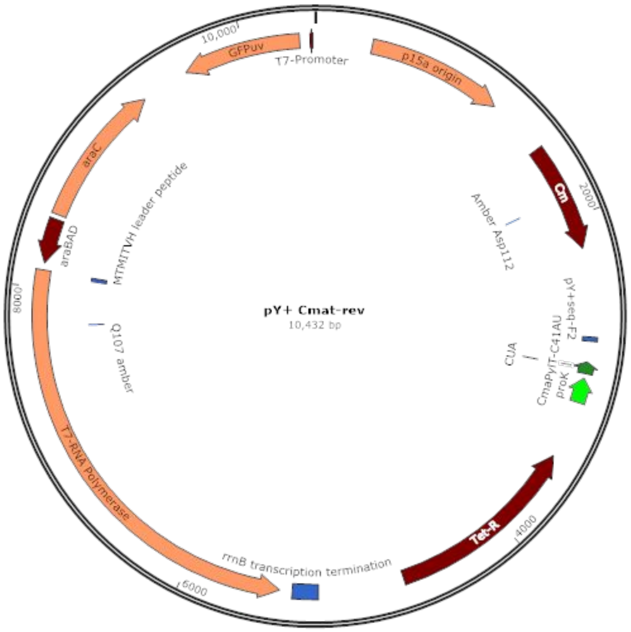

pBK-CmaPylRS plasmid sequence

ctcgggttgtcagcctgtcccgcttataagatcatacgccgttatacgttgtttacgctttgaggaatcccatATGACGGTAAAGT
ACACAGATGCTCAGATCCAACGTCTTCGCGAATACGGAAATGGGACCTATGAACAGAAGG
TGTTTGAGGATTTGGCCTCCCGCGACGCGGCTTTCTCGAAAGAGATGTCCGTTGCATCGA
CGGACAATGAAAAAAAGATCAAAGGCATGATTGCAAACCCTTCTCGTCATGGCTTAACAC
AATTAATGAATGATATCGCAGATGCTTTGGTTGCTGAAGGCTTTATCGAGGTCCGTACTCC
TATCTTTATTTCCAAAGACGCTTTGGCCCGCATGACAATCACGGAAGACAAACCCTTGTTT
AAGCAGGTTTTCTGGATTGATGAAAAACGTGCCTTACGTCCAATGCTTGCTCCAAACCTTT
ACTCTGTCATGCGTGACTTGCGTGACCACACGGACGGGCCTGTAAAGATtTTCGAGATGG
GATCTTGCTTTCGTAAAGAATCGCACTCGGGGATGCACCTTGAGGAGTTCACTATGTTGA
ATTTAGTTGATATGGGACCTCGCGGGGATGCTACGGAAGTTCTGAAGAACTATATTAGCG
TCGTTATGAAGGCGGCTGGCCTGCCAGACTACGATTTAGTGCAGGAAGAATCGGACGTG
TACAAGGAAACCATTGACGTCGAAATCAATGGGCAAGAGGTATGTAGCGCCGCAGTGGG
ACCACATTACCTTGATGCAGCTCACGATGTACATGAGCCGTGGTCGGGGGCGGGGTTTG
GTTTAGAGCGCCTTCTGACGATCCGTGAAAAATACTCTACCGTGAAGAAGGGCGGTGCTA
GTATCAGTTATTTGAACGGGGCCAAGATCAACTGActgcagtttcaaacgctaaattgcctgatgcgctacgc
ttatcaggcctacatgatctctgcaatatattgagtttgcgtgcttttgtaggccggataaggcgttcacgccgcatccggcaagaaaca
gcaaacaatccaaaacgccgcgttcagcggcgttttttctgcttttcttcgcgaattaattccgcttcgcacatgtgagcaaaaggccag
caaaaggccaggaaccgtaaaaaggccgcgttgctggcgtttttccataggctccgcccccctgacgagcatcacaaaaatcgac
gctcaagtcagaggtggcgaaacccgacaggactataaagataccaggcgtttccccctggaagctccctcgtgcgctctcctgttc
cgaccctgccgcttaccggatacctgtccgcctttctcccttcgggaagcgtggcgctttctcatagctcacgctgtaggtatctcagttc
ggtgtaggtcgttcgctccaagctgggctgtgtgcacgaaccccccgttcagcccgaccgctgcgccttatccggtaactatcgtcttg
agtccaacccggtaagacacgacttatcgccactggcagcagccactggtaacaggattagcagagcgaggtatgtaggcggtg
ctacagagttcttgaagtggtggcctaactacggctacactagaaggacagtatttggtatctgcgctctgctgaagccagttaccttcg

gaaaaagagttggtagctcttgatccggcaaacaaaccaccgctggtagcggtggtttttttgtttgcaagcagcagattacgcgcag
aaaaaaaggatctcaagaagatcctttgatcttttctacggggtctgacgctcagtggaacgaaaactcacgttaagggattttggtca
tgagttgtgtctcaaaatctctgatgttacattgcacaagataaaaatatatcatcatgaacaataaaactgtctgcttacataaacagta
atacaaggggtgttatgagccatattcaacgggaaacgtcttgctcgaggccgcgattaaattccaacatggatgctgatttatatggg
tataaatgggctcgcgataatgtcgggcaatcaggtgcgacaatctatcgattgtatgggaagcccgatgcgccagagttgtttctga
aacatggcaaaggtagcgttgccaatgatgttacagatgagatggtcagactaaactggctgacggaatttatgcctcttccgaccat
caagcattttatccgtactcctgatgatgcatggttactcaccactgcgatccccgggaaaacagcattccaggtattagaagaatatc
ctgattcaggtgaaaatattgttgatgcgctggcagtgttcctgcgccggttgcattcgattcctgtttgtaattgtccttttaacagcgatcg
cgtatttcgtctcgctcaggcgcaatcacgaatgaataacggtttggttgatgcgagtgattttgatgacgagcgtaatggctggcctgtt
gaacaagtctggaaagaaatgcataagcttttgccattctcaccggattcagtcgtcactcatggtgatttctcacttgataaccttattttt
gacgaggggaaattaataggttgtattgatgttggacgagtcggaatcgcagaccgataccaggatcttgccatcctatggaactgc
ctcggtgagttttctccttcattacagaaacggctttttcaaaaatatggtattgataatcctgatatgaataaattgcagtttcatttgatgct
cgatgagtttttctaatcagaattggttaattggttgtaacactggcagagcattacgctgacttgacgggacggcggctttgttgaataa
atcgaacttttgctgagttgaaggatc

pY+-CmaPylT-C41AU

gcccttccggctggctggtttattgctgataaatctggagccggtgagcgtgggtctcgcggtatcattgcagcactggggccagatgg
taagccctcccgtatcgtagttatctacacgacggggagtcaggcaactatggatgaacgaaatagacagatcgctgagataggtg
cctcactgattaagcattggtaactgtcagaccaagtttactcatatatactttagattgatttaaaacttcatttttaatttaaaaggatctag
gtgaagatcctttttgataatctcatgaccaaaatcccttaacgtgagttttcgttccactgagcgtcagaccccttaataagatgatcttct
tgagatcgttttggtctgcgcgtaatctcttgctctgaaaacgaaaaaaccgccttgcagggcggtttttcgaaggttctctgagctacca
actctttgaaccgaggtaactggcttggaggagcgcagtcaccaaaacttgtcctttcagtttagccttaaccggcgcatgacttcaag
actaactcctctaaatcaattaccagtggctgctgccagtggtgcttttgcatgtctttccgggttggactcaagacgatagttaccggat
aaggcgcagcggtcggactgaacggggggttcgtgcatacagtccagcttggagcgaactgcctacccggaactgagtgtcagg
cgtggaatgagacaaacgcggccataacagcggaatgacaccggtaaaccgaaaggcaggaacaggagagcgcacgagg
gagccgccagggggaaacgcctggtatctttatagtcctgtcgggtttcgccaccactgatttgagcgtcagatttcgtgatgcttgtca
ggggggcggagcctatggaaaaacggctttgccgcggccctctcacttccctgttaagtatcttcctggcatcttccaggaaatctccg
ccccgttcgtaagccatttccgctcgccgcagtcgaacgaccgagcgtagcgagtcagtgagcgaggaagcggaatatatcctgta
tcacatattctgctgacgcaccggtgcagcctttttctcctgccacatgaagcacttcactgacaccctcatcagtgccaacatagtaa
gccagtatacactccgctagcgctgatgtccggcggtgcttttgccgttacgcaccaccccgtcagtagctgaacaggagggacag
ctgatagaaacagaagccactggagcacctcaaaaacaccatcatacactaaatcagtaagttggcagcatcacccgacgcactt
tgcgccgaataaatacctgtgacggaagatcacttcgcagaataaataaatcctggtgtccctgttgataccgggaagccctgggcc
aacttttggcgaaaatgagacgttgatcggcacgtaagaggttccaactttcaccataatgaaataagatcactaccgggcgtattttt
gagttatcgagattttcaggagctaaggaagctaaaatggagaaaaaaatcactggatataccaccgttgatatatcccaatggcat
cgtaaagaacattttgaggcatttcagtcagttgctcaatgtacctataaccagaccgttcagctggatattacggcctttttaaagaccg
taaagaaaaataagcacaagttttatccggcctttattcacattcttgcccgcctgatgaatgctcatccggaattccgtatggcaatga
aagacggtgagctggtgatatgggatagtgttcacccttgttacaccgttttccatgagcaaactgaaacgttttcatcgctctggagtg
aataccactaggatttccggcagtttctacacatatattcgcaagatgtggcgtgttacggtgaaaacctggcctatttccctaaagggtt
tattgagaatatgtttttcgtctcagccaatccctgggtgagtttcaccagttttgatttaaacgtggccaatatggacaacttcttcgcccc
cgttttcaccatgggcaaatattatacgcaaggcgacaaggtgctgatgccgctggcgattcaggttcatcatgccgtttgtgatggctt
ccatgtcggcagaatgcttaatgaattacaacagtactgcgatgagtggcagggcggggcgtaatttttttaaggcagttattggtgcc
cttaaacgcctggttgctacgcctgaataagtgataataagcggatgaatggcagaaattcgaaagcaaattcgacccggtcgtcg

gttcagggcagggtcgttaaatagccgcttatgtctattgctggtttaccggtttattgactaccggaagcagtgtgaccgtgtgcttctca
aatgcctgaggccagtttgctcaggctctccccgtggaggtaataattgacgatatgatcatttattctgcctcccagagcatgataaaa
acggttagcgcttcgttaatacagatgtaggtgttccacagggtagccagcagcatcctgcgatgcagatccggaacataatggtgc
agggcgcttgtttcggcgtgggtatggtggcaggccccgtggccgggggactgttgggcgctgccggcacctgtcctacgagttgca
tgataaagaagacagtcataagtgcggcgacgatagtcatgccccgcgcccaccggaaggagctaccggcagcggtgcggact
gttgtaactcagaataagaaatgaggccgctcatggcgttctgttgcccgtctcactggtgaaaagaaaaacaaccctggcgccgct
tctttgagcgaacgatcaaaaataagtggcgcagatctggcttttcgaattTGGCGAGAGACCGGGGCGTCGAACC
CCGCTATGCTAGGTTTTAGAGACCCGCTGGTCGCCGGACCGTCCCCCaatgcggggcgcatctta
ctgcgcagatacgccctcgtcaatcccttaatagcaaaatgccttttgatcggcgagaaagtcagcaggccgcttagttagccgtgca
ctgatcatatcgtcaattattacctccacggggagagcctgagcaaactggcctcaggcatttgagaagcacaagatctagcttggc
actggccgtcgttttacaacgtcgtgactgggaaaaccctggcgttacccaacttaatcgccttgcagcacatccccctttcgccagac
gctctcccttatgcgactcctgcattaggaagcagcccagtagtaggttgaggccgttgagcaccgccgccgcaaggaatggtgcat
gcaaggagcccgagatgcgccgcgtgcggctgctggagatggcggacgcgatggatatgttctgccaagggttggtttgcgcattc
acagttctccgcaagaattgattggctccaattcttggagtggtgaatccgttagcgaggtgccgccggcttccattcaggtcgaggtg
gcccggctccatgcaccgcgacgcaacgcggggaggcagacaaggtatagggcggcgcctacaatccatgccaacccgttcca
tgtgctcgccgaggcggcataaatcgccgtgacgatcagcggtccaatgatcgaagttaggctggtaagagccgcgagcgatcctt
gaagctgtccctgatggtcgtcatctacctgcctggacagcatggcctgcaacgcgggcatcccgatgccgccggaagcgagaag
aatcataatggggaaggccatccagcctcgcgtcgcgaacgccagcaagacgtagcccagcgcgtcggccgccatgccggcg
ataatggcctgcttctcgccgaaacgtttggtggcgggaccagtgacgaaggcttgagcgagggcgtgcaagattccgaataccgc
aagcgacaggccgatcatcgtcgcgctccagcgaaagcggtcctcgccgaaaatgacccagagcgctgccggcacctgtcctac
gagttgcatgataaagaagacagtcataagtgcggcgacgatagtcatgccccgcgcccaccggaaggagctgactgggttgaa
ggctctcaagggcatcggtcgacgctctcccttatgcgactcctgcattaggaagcagcccagtagtaggttgaggccgttgagcac
cgccgccgcaaggaatggtgcatgcaaggagatggcgcccaacagtcccccggccacggggcctgccaccatacccacgccg
aaacaagcgctcatgagcccgaagtggcgagcccgatcttccccatcggtgatgtcggcgatataggcgccagcaaccgcacct
gtggcgccggtgatgccggccacgatgcgtccggcgtagaggatccacaggacgggtgtggtcgccatgatcgcgtagtcgatag
tggctccaagtagcgaagcgagcaggactgggcggcggccaaagcggtcggacagtgctccgagaacgggtgcgcatagaaa
ttgcatcaacgcatatagcgctagcagcacgccatagtgactggcgatgctgtcggaatggacgatatcccgcaagaggcccggc
agtaccggcataaccaagcctatgcctacagcatccagggtgacggtgccgaggatgacgatgagcgcattgttagatttcataca
cggtgcctgactgcgttagcaatttaactgtgataaactaccgcattaaagcttatcgatgataagctgtcaaacatgagaattacaac
ttatatcgtatggggctgacttcaggtgctacattgctcaaagatgcaggggtaaaagctaaccgcatctttaccgacaaggcatccg
gcagttcaacagatcgggaagggctggatttgctgaggatgaaggtggaggaaggtgatgtcattctggtgaagaagctcgaccgt
cttggccgcgacaccgccgacatgatccaactgataaaagagtttgatgctcagggtgtagcggttcggtttattgacgacgggatca
gtaccgacggtgatatggggcaaatggtggtcaccatcctgtcggctgtggcacaggctgaacgccggaggatcctccgggccgtt
gcttcgcaacgttcaaatccgctcccggcggatttgtcctactcaggagagcgttcaccgacaaacaacagataaaacgaaaggc
ccagtctttcgactgagcctttcgttttatttgatgcctggcagttccctactctcgcatggggagaccccacactaccatcggcgctacg
gcgtttcacttctgagttcggcatggggtcaggtgggaccaccgcgctactgccgccaggcaaattctgttttatcagaccgcttctgcg
ttctgatttaatctgtatcaggctgaaaatcttctctcatccgacgtcttaggcgaaggcgaagtccgactctaagatgtcacggaggttc
aagttacctttagccggaagtgctggcattttgtccaattgagactcgtgcaactggtcagcgaactggtcgtagaaatcagccagtac
atcacaagactcatatgtgtcaaccatagtttcgcgcactgctttgaacaggttcgcagcgtcagccggaatggtaccgaaggagtc
gtgaatcagtgcaaaagattcgattccgtacttctcgtgtgcccacactacagtcttacgaaggtggctaccgtcttggctgtgtacaaa
gttaggagcgataccagactcctgtttgtgtgcatcaatctcgctatctttgttggtgttaatggtaggctgtaagcggaactgaccgagg
aacatcaggttcaagcgcgtctgaataggcttcttgtattcctgccacacagggaaaccatcaggagttacccaatgcacagcgca

acgcttgcgaagaatctctccagtcttcttatctttgacctcagcagccagcagcttagcagcagacttaagccagttcattgcttcaac
cgcagctaccaccgtcacgctcacagattcccaaatcagcttagccatgtatccagcagcctgattcggctgagtgaacatcagacc
cttgccggaatcaatagctggctgaatggtatcttccagcacttgttgacggaagccgaactctttggacccgtaagccagcgtcatg
actgaacgcttagtcacactgcgagtaacaccgtaagccagccattgaccagccagtgccttagtgcccagcttgactttctcagag
atttcaccagtgttctcatcggtcacggtaactacttcgttatcggtcccattgattgcgtctgcttgtagaatctcgttgactttcttagcaac
aatcccgtagatgtcctgaacggtttcactaggaagcaagttaaccgcgcgaccacctacctcatctcggagcatcgcggagaagt
gctggatgccagagcaagacccgtcaaacgccagcggaagggagcagttatagctcaggccgtggtgctgtaccccagcgtact
caaagcagaacgcaaggaagcagaacggagaatcttgctcagcccaccaagtgttctccagtggagacttagcgcaagccatg
atgttctcgtggttttcctcaatgaacttgatgcgctcagggaacggaaccttatcgacacccgcacagtttgcaccgtggattttcagcc
agtagtaaccttccttaccgattggtttacctttcgccagcgtaagcagtcctttggtcatatcgttaccttgcgggttgaacattgacaca
gcgtaaacacgaccgcgccagtccatgttgtaagggaaccagatggccttatggttagcaaacttattggcttgctcaagcatgaact
caaggctgatacggcgagacttgcgagccttgtccttgcggtacacagcagcggcagcacgtttccacgcggtgagagcctcagg
attcatgtcgatgtcttccggtttcatcgggagttcttcacgctcaatcgcagggatgtcctcgaccggacaatgcttccacttggtgatta
cgttggcgaccgctaggactttcttgttgattttccatgcggtgttttgcgcaatgttaatcgctttgtacacctcaggcatgtaaacgtcttc
gtagcgcatcagtgctttcttactgtgagtacgcaccagcgccagaggacgacgaccgttagcccaatagccaccaccagtaatgc
cagtccacggcttaggaggaactacgcaaggttggaacatcggagagatgccagccagcgcacctgcacgggttgcgatagcct
cagcgtattcaggtgcgagttcgatagtctcagagtcttgacctactacgccagcattttggcggtgtaagctaaccattccggttgact
caatgagcatctcgatgcagcgtactcctacatgaatagagtcttccttatgccacgaagaccacgcctcgccaccgagtagaccct
tagagagcatgtcagcctcgacaacttgcataaatgctttcttgtagacgtgccctacgcgcttgttgagttgttcctcaacgtttttcttga
agtgcttagcttcaaggtcacggatacgaccgaagcgagcctcgtcctcaatggcccgaccgattgcgcttgctacagcctgaacg
gttgtattgtcagcactggttaggcaagccagagtggtcttaatggtgatgtacgctacggcttccggcttgatttcctacaggaactgg
aaggctgtcgggcgcttgccgcgcttagctttcacttcctcaaaccagtcgttgatgcgtgcaatcatcttagggagtagggtagtgatg
agaggcttggcggcagcgttatccgcaacctcaccagctttaagttgacgctcaaacatcttgcggaagcgtgcttcacccatctcgt
aagactcatgctcaagggccaactgttcgcgagctaaacgctcaccgtaatggtcagccagagtgttgaacgggatagcagccag
ttcgatgtcagagaagtcgttcttagcaatgttaatggtattctagtgcacggtaatcatggtcatggttaattcctcctgttagcccaaaa
aacgggtatggagaaacagtagagagttgcgataaaaagcgtcaggtaggatccgctaatcttatggataaaaatgctatggcata
gcaaagtgtgacgccgtgcaaataatcaatgtggacttttctgccgtgattatagacacttttgttacgcgtttttgtcatggctttggtccc
gctttgttacagaatgcttttaataagcggggttaccggtttggttagcgagaagagccagtaaaagacgcagtgacggcaatgtctg
atgcaatatggacaattggtttcttctctgaatggcgggagtatgaaaagtatggctgaagcgcaaaatgatcccctgctgccgggat
actcgtttaatgcccatctggtggcgggtttaacgccgattgaggccaacggttatctcgatttttttatcgaccgaccgctgggaatgaa
aggttatattctcaatctcaccattcgcggtcaggggggtggtgaaaaatcagggacgagaatttgtttgccgaccgggtgatattttgct
gttcccgccaggagagattcatcactacggtcgtcatccggaggctcgcgaatggtatcaccagtgggtttactttcgtccgcgcgcct
actggcatgaatggcttaactggccgtcaatatttgccaatacggggttctttcgcccggatgaagcgcaccagccgcatttcagcga
cctgtttgggcaaatcattaacgccgggcaaggggaagggcgctattcggagctgctggcgataaatctgcttgagcaattgttactg
cggcgcatggaagcgattaacgagtcgctccatccaccgatggataatcgggtacgcgaggcttgtcagtacatcagcgatcacct
ggcagacagcaattttgatatcgccagcgtcgcacagcatgtttgcttgtcgccgtcgcgtctgtcacatcttttccgccagcagttagg
gattagcgtcttaagctggcgcgaggaccaacgtatcagccaggcgaagctgcttttgagcaccacccggatgcctatcgccaccg
tcggtcgcaatgttggttttgacgatcaactctatttctcgcgggtatttaaaaaatgcaccggggccagcccgagcgagttccgtgcc
ggttgtgaagaaaaagtgaatgatgtagccgtcaagttgtcataattggtaacgaatcagacaattgacggcttgacggagtagcat
agggtttgcagaatccctgcttcgtccatttgacaggcacattatgcatgccgcttcgccttcgcgcgcgaattgatctgctgcctcgcg
cgtttcggtgatgacggtgaaaacctctgacacatgcagctcccggagacggtcacagcctgcagcaaaaaacccctcaagacc
cgtttagaggccccaaggggttatgctagttattgctcagcggtggcagcagccaactcagcttcctttcgggctttgttatttgtagagct

catccatgccatgtgtaatcccagcagcagttacaaactcaagaaggaccatgtggtcacgcttttcgttgggatctttcgaaagggc
agattgtgtcgacaggtaatggttgtctggtaaaaggacagggccatcgccaattggagtattttgttgataatggtctgctagttgaac
ggatccatcttcaatgttgtggcgaattttgaagttagctttgattccattcttttgtttgtctgccgtgatgtatacattgtgtgagttatagttgt
actcgagtttgtgtccgagaatgtttccatcttctttaaaatcaataccttttaactcgatacgattaacaagggtatcaccttcaaacttga
cttcagcacgcgtcttgtagttcccgtcatctttgaaagatatagtgcgttcctgtacataaccttcgggcatggcactcttgaaaaagtc
atgccgtttcatatgatccggataacgggaaaagcattgaacaccataagagaaagtagtgacaagtgttggccatggaacaggt
agttttccagtagtgcaaataaatttaagggtaagttttccgtatgttgcatcaccttcaccctctccactgacagaaaatttgtgcccatta
acatcaccatctaattcaacaagaattgggacaactccagtgaaaagttcttctcctttactcatatgtatatctccttcttaaagttaaac
aaaattatttctagagggaaaccgttgtggtctccctatagtgagtcgtattaatttcgcgggatcg

Figure S6. Plasmid maps for pBK-CmaPylS and pY+-CmaPylT-C41AU.

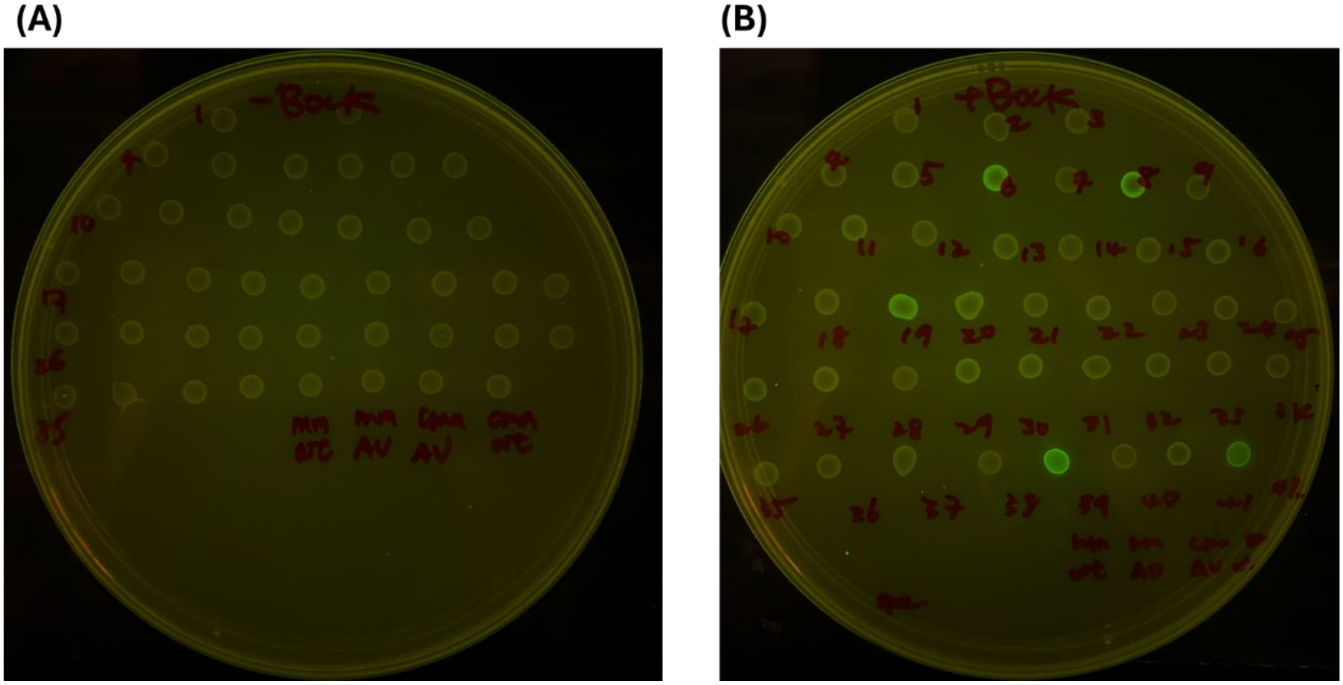

Figure S7. Spotting plates for fluorescence screening. The last 4 spots are controls for MmPylS/MmPylT, MmPylS/CmaPylT-C41AU, CmaPylS/CmaPylT-C41AU and CmaPylS/CmaPylT respectively.

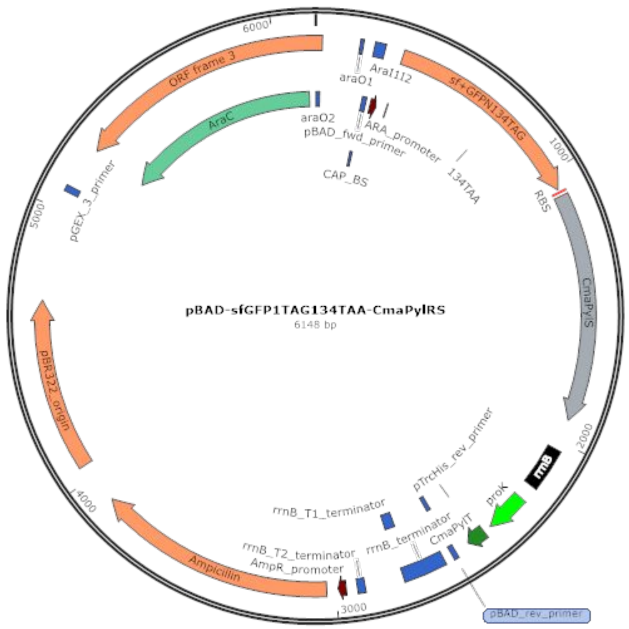

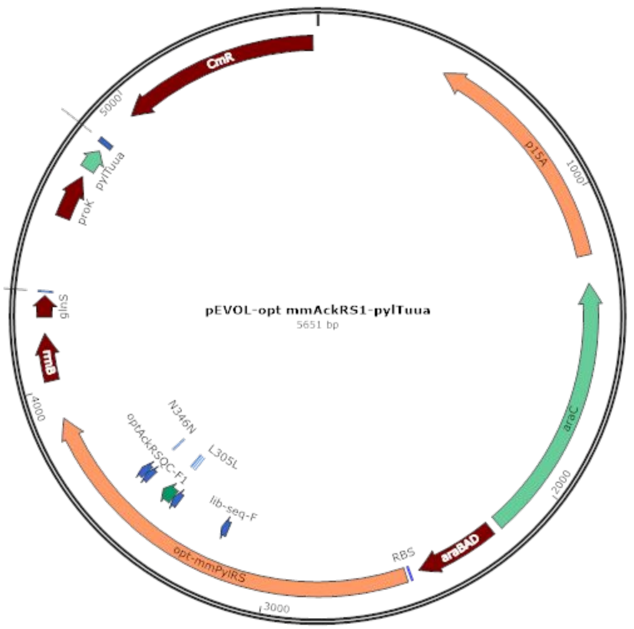

pBAD-sfGFP-CmaPylRS

aagaaaccaattgtccatattgcatcagacattgccgtcactgcgtcttttactggctcttctcgctaaccaaaccggtaaccccgcttat
taaaagcattctgtaacaaagcgggaccaaagccatgacaaaaacgcgtaacaaaagtgtctataatcacggcagaaaagtcc
acattgattatttgcacggcgtcacactttgctatgccatagcatttttatccataagattagcggatcctacctgacgctttttatcgcaact
ctctactgtttctccatacccgtttttttgggctaacaggaggaattaaccatggcgtaggttagcaaaggtgaagaactgtttaccggcg
ttgtgccgattctggtggaactggatggtgatgtgaatggccataaatttagcgttcgtggcgaaggcgaaggtgatgcgaccaacg
gtaaactgaccctgaaatttatttgcaccaccggtaaactgccggttccgtggccgaccctggtgaccaccctgacctatggcgttca
gtgctttagccgctatccggatcatatgaaacgccatgatttctttaaaagcgcgatgccggaaggctatgtgcaggaacgtaccatta
gcttcaaagatgatggcacctataaaacccgtgcggaagttaaatttgaaggcgataccctggtgaaccgcattgaactgaaaggt
attgattttaaagaataaggcaacattctgggtcataaactggaatataatttcaacagccataatgtgtatattaccgccgataaaca
gaaaaatggcatcaaagcgaactttaaaatccgtcacaacgtggaagatggtagcgtgcagctggcggatcattatcagcagaat
accccgattggtgatggcccggtgctgctgccggataatcattatctgagcacccagagcgttctgagcaaagatccgaatgaaaa
acgtgatcatatggtgctgctggaatttgttaccgccgcgggcattacccacggtatggatgaactgtataaaggcagccaccatcat
catcaccattgaagctcgagaggaggaattactagtATGACGGTAAAGTACACAGATGCTCAGATCCAACG
TCTTCGCGAATACGGAAATGGGACCTATGAACAGAAGGTGTTTGAGGATTTGGCCTCCCG
CGACGCGGCTTTCTCGAAAGAGATGTCCGTTGCATCGACGGACAATGAAAAAAAGATCAA
AGGCATGATTGCAAACCCTTCTCGTCATGGCTTAACACAATTAATGAATGATATCGCAGAT
GCTTTGGTTGCTGAAGGCTTTATCGAGGTCCGTACTCCTATCTTTATTTCCAAAGACGCTT
TGGCCCGCATGACAATCACGGAAGACAAACCCTTGTTTAAGCAGGTTTTCTGGATTGATG
AAAAACGTGCCTTACGTCCAATGCTTGCTCCAAACCTTTACTCTGTCATGCGTGACTTGCG
TGACCACACGGACGGGCCTGTAAAGATtTTCGAGATGGGATCTTGCTTTCGTAAAGAATC
GCACTCGGGGATGCACCTTGAGGAGTTCACTATGTTGAATTTAGTTGATATGGGACCTCG
CGGGGATGCTACGGAAGTTCTGAAGAACTATATTAGCGTCGTTATGAAGGCGGCTGGCC
TGCCAGACTACGATTTAGTGCAGGAAGAATCGGACGTGTACAAGGAAACCATTGACGTCG
AAATCAATGGGCAAGAGGTATGTAGCGCCGCAGTGGGACCACATTACCTTGATGCAGCT
CACGATGTACATGAGCCGTGGTCGGGGGCGGGGTTTGGTTTAGAGCGCCTTCTGACGAT

CCGTGAAAAATACTCTACCGTGAAGAAGGGCGGTGCTAGTATCAGTTATTTGAACGGGGC
CAAGATCAACTGAgtcgactgagtttaaacggtctccagcttggctgttttggcggatgagagaagattttcagcctgatacag
attaaatcagaacgcagaagcggtctgataaaacagaatttgcctggcggcagtagcgcggtggtcccacctgaccccatgccga
actcagaagtgaaacgccgtagcgccgatggtagtgtggggtctccccatgcgagagtagggaactgccaggcatcaaataaaa
cgaaaggctcagtcgaaagactgggccttgtttgtgagctcacatgtgagcaccggtttattgactaccggaagcagtgtgaccgtgt
gcttctcaaatgcctgaggccagtttgctcaggctctccccgtggaggtaataattgacgatatgatcagtgcacggctaactaagcg
gcctgctgactttctcgccgatcaaaaggcattttgctattaagggattgacgagggcgtatctgcgcagtaagatgcgccccgcatt
GGGGGACGGTCCGGCGACCAGCGGGTCTCTAAAACCTAGCCAGCGGGGTTCGACGCCC
CGGTCTCTCGCCAaattcgaaaagcctgaagcttggctgttttggcggatgagagaagattttcagcctgatacagattaaat
cagaacgcagaagcggtctgataaaacagaatttgcctggcggcagtagcgcggtggtcccacctgaccccatgccgaactcag
aagtgaaacgccgtagcgccgatggtagtgtggggtctccccatgcgagagtagggaactgccaggcatcaaataaaacgaaa
ggctcagtcgaaagactgggcctttcgttttatctgttgtttgtcggtgaacgctctcctgagtaggacaaatccgccgggagcggatttg
aacgttgcgaagcaacggcccggagggtggcgggcaggacgcccgccataaactgccaggcatcaaattaagcagaaggcc
atcctgacggatggcctttttgcgtttctacaaactcttttgtttatttttctaaatacattcaaatatgtatccgctcatgagacaataaccctg
ataaatgcttcaataatattgaaaaaggaagagtatgagtattcaacatttccgtgtcgcccttattcccttttttgcggcattttgccttcct
gtttttgctcacccagaaacgctggtgaaagtaaaagatgctgaagatcagttgggtgcacgagtgggttacatcgaactggatctca
acagcggtaagatccttgagagttttcgccccgaagaacgttttccaatgatgagcacttttaaagttctgctatgtggcgcggtattatc
ccgtgttgacgccgggcaagagcaactcggtcgccgcatacactattctcagaatgacttggttgagtactcaccagtcacagaaa
agcatcttacggatggcatgacagtaagagaattatgcagtgctgccataaccatgagtgataacactgcggccaacttacttctgac
aacgatcggaggaccgaaggagctaaccgcttttttgcacaacatgggggatcatgtaactcgccttgatcgttgggaaccggagct
gaatgaagccataccaaacgacgagcgtgacaccacgatgcctgtagcaatggcaacaacgttgcgcaaactattaactggcga
actacttactctagcttcccggcaacaattaatagactggatggaggcggataaagttgcaggaccacttctgcgctcggcccttccg
gctggctggtttattgctgataaatctggagccggtgagcgtgggtctcgcggtatcattgcagcactggggccagatggtaagccctc
ccgtatcgtagttatctacacgacggggagtcaggcaactatggatgaacgaaatagacagatcgctgagataggtgcctcactga
ttaagcattggtaactgtcagaccaagtttactcatatatactttagattgatttaaaacttcatttttaatttaaaaggatctaggtgaagat
cctttttgataatctcatgaccaaaatcccttaacgtgagttttcgttccactgagcgtcagaccccgtagaaaagatcaaaggatcttct
tgagatcctttttttctgcgcgtaatctgctgcttgcaaacaaaaaaaccaccgctaccagcggtggtttgtttgccggatcaagagcta
ccaactctttttccgaaggtaactggcttcagcagagcgcagataccaaatactgtccttctagtgtagccgtagttaggccaccacttc
aagaactctgtagcaccgcctacatacctcgctctgctaatcctgttaccagtggctgctgccagtggcgataagtcgtgtcttaccgg
gttggactcaagacgatagttaccggataaggcgcagcggtcgggctgaacggggggttcgtgcacacagcccagcttggagcg
aacgacctacaccgaactgagatacctacagcgtgagctatgagaaagcgccacgcttcccgaagggagaaaggcggacagg
tatccggtaagcggcagggtcggaacaggagagcgcacgagggagcttccagggggaaacgcctggtatctttatagtcctgtcg
ggtttcgccacctctgacttgagcgtcgatttttgtgatgctcgtcaggggggcggagcctatggaaaaacgccagcaacgcggcctt
tttacggttcctggccttttgctggccttttgctcacatgttctttcctgcgttatcccctgattctgtggataaccgtattaccgcctttgagtga
gctgataccgctcgccgcagccgaacgaccgagcgcagcgagtcagtgagcgaggaagcggaagagcgcctgatgcggtattt
tctccttacgcatctgtgcggtatttcacaccgcatatggtgcactctcagtacaatctgctctgatgccgcatagttaagccagtataca
ctccgctatcgctacgtgactgggtcatggctgcgccccgacacccgccaacacccgctgacgcgccctgacgggcttgtctgctcc
cggcatccgcttacagacaagctgtgaccgtctccgggagctgcatgtgtcagaggttttcaccgtcatcaccgaaacgcgcgagg
cagcagatcaattcgcgcgcgaaggcgaagcggcatgcataatgtgcctgtcaaatggacgaagcagggattctgcaaaccctat
gctactccgtcaagccgtcaattgtctgattcgttaccaattatgacaacttgacggctacatcattcactttttcttcacaaccggcacgg
aactcgctcgggctggccccggtgcatttttaaatacccgcgagaaatagagttgatcgtcaaaaccaacattgcgaccgacggtg
gcgataggcatccgggtggtgctcaaaagcagcttcgcctggctgatacgttggtcctcgcgccagcttaagacgctaatccctaact

gctggcggaaaagatgtgacagacgcgacggcgacaagcaaacatgctgtgcgacgctggcgatatcaaaattgctgtctgcca
ggtgatcgctgatgtactgacaagcctcgcgtacccgattatccatcggtggatggagcgactcgttaatcgcttccatgcgccgcagt
aacaattgctcaagcagatttatcgccagcagctccgaatagcgcccttccccttgcccggcgttaatgatttgcccaaacaggtcgc
tgaaatgcggctggtgcgcttcatccgggcgaaagaaccccgtattggcaaatattgacggccagttaagccattcatgccagtagg
cgcgcggacgaaagtaaacccactggtgataccattcgcgagcctccggatgacgaccgtagtgatgaatctctcctggcgggaa
cagcaaaatatcacccggtcggcaaacaaattctcgtccctgatttttcaccaccccctgaccgcgaatggtgagattgagaatataa
cctttcattcccagcggtcggtcgataaaaaaatcgagataaccgttggcctcaatcggcgttaaacccgccaccagatggcatta
aacgagtatcccggcagcaggggatcattttgcgcttcagccatacttttcatactcccgccattcagag

pEVOL-AcKRS1-MmPylT-UUA

tcctgaaaatctcgataactcaaaaaatacgcccggtagtgatcttatttcattatggtgaaagttggaacctcttacgtgccgatcaac
gtctcattttcgccaaaagttggcccagggcttcccggtatcaacagggacaccaggatttatttattctgcgaagtgatcttccgtcac
aggtatttattcggcgcaaagtgcgtcgggtgatgctgccaacttactgatttagtgtatgatggtgtttttgaggtgctccagtggcttctgt
ttctatcagctgtccctcctgttcagctactgacggggtggtgcgtaacggcaaaagcaccgccggacatcagcgctagcggagtgt
atactggcttactatgttggcactgatgagggtgtcagtgaagtgcttcatgtggcaggagaaaaaaggctgcaccggtgcgtcagc
agaatatgtgatacaggatatattccgcttcctcgctcactgactcgctacgctcggtcgttcgactgcggcgagcggaaatggcttac
gaacggggcggagatttcctggaagatgccaggaagatacttaacagggaagtgagagggccgcggcaaagccgtttttccata
ggctccgcccccctgacaagcatcacgaaatctgacgctcaaatcagtggtggcgaaacccgacaggactataaagataccagg
cgtttccccctggcggctccctcgtgcgctctcctgttcctgcctttcggtttaccggtgtcattccgctgttatggccgcgtttgtctcattcca
cgcctgacactcagttccgggtaggcagttcgctccaagctggactgtatgcacgaaccccccgttcagtccgaccgctgcgccttat
ccggtaactatcgtcttgagtccaacccggaaagacatgcaaaagcaccactggcagcagccactggtaattgatttagaggagtt
agtcttgaagtcatgcgccggttaaggctaaactgaaaggacaagttttggtgactgcgctcctccaagccagttacctcggttcaaa
gagttggtagctcagagaaccttcgaaaaaccgccctgcaaggcggttttttcgttttcagagcaagagattacgcgcagaccaaaa
cgatctcaagaagatcatcttattaatcagataaaatatttctagatttcagtgcaatttatctcttcaaatgtagcacctgaagtcagccc
catacgatataagttgtaattctcatgtttgacagcttatcatcgataagcttggtacccaattatgacaacttgacggctacatcattcact
ttttcttcacaaccggcacggaactcgctcgggctggccccggtgcattttttaaatacccgcgagaaatagagttgatcgtcaaaacc
aacattgcgaccgacggtggcgataggcatccgggtggtgctcaaaagcagcttcgcctggctgatacgttggtcctcgcgccagct
taagacgctaatccctaactgctggcggaaaagatgtgacagacgcgacggcgacaagcaaacatgctgtgcgacgctggcgat
atcaaaattgctgtctgccaggtgatcgctgatgtactgacaagcctcgcgtacccgattatccatcggtggatggagcgactcgttaa
tcgcttccatgcgccgcagtaacaattgctcaagcagatttatcgccagcagctccgaatagcgcccttccccttgcccggcgttaatg
atttgcccaaacaggtcgctgaaatgcggctggtgcgcttcatccgggcgaaagaaccccgtattggcaaatattgacggccagtta
agccattcatgccagtaggcgcgcggacgaaagtaaacccactggtgataccattcgcgagcctccggatgacgaccgtagtgat
gaatctctcctggcgggaacagcaaaatatcactcggtcggcaaacaaattctcgtccctgatttttcaccaccccctgaccgcgaat
ggtgagattgagaatataacctttcattcccagcggtcggtcgataaaaaaatcgagataaccgttggcctcaatcggcgttaaaccc
gccaccagatgggcattaaacgagtatcccggcagcaggggatcattttgcgcttcagccatacttttcatactcccgccattcagag
aagaaaccaattgtccatattgcatcagacattgccgtcactgcgtcttttactggctcttctcgctaaccaaaccggtaaccccgcttat
taaaagcattctgtaacaaagcgggaccaaagccatgacaaaaacgcgtaacaaaagtgtctataatcacggcagaaaagtcc
acattgattatttgcacggcgtcacactttgctatgccatagcatttttatccataagattagcggatcctacctgacgctttttatcgcaact
ctctactgtttctccatacccgtttttttgggctaacaggaggaattactagtatggataagaaaccgctgaatactctgatttctgcaactg
gtctgtggatgagccgtaccggcaccatccacaagatcaaacaccacgaggtttcccgtagcaaaatctacatcgaaatggcgtgc
ggtgaccacctggtggtaaacaactcccgttcttctcgtactgcacgtgctctgcgccaccacaagtaccgtaagacctgcaagcgct
gtcgcgtgtctgatgaagacctgaacaaattcctgactaaagcgaacgaagatcagacttctgtgaaggtgaaagttgtttctgcccc

aacccgcaccaagaaagcgatgccgaagtccgttgcacgcgctccgaaaccgctggagaacaccgaagccgcacaggccca
gccgtctggttctaagttttctccggcaatcccggtttctactcaggagtctgtgtctgtgccagcttctgttagcacttctatttcctctatcag
cactggtgcgactgcgtccgctctggtaaaaggtaacactaacccgatcaccagcatgtctgctccggttcaggcttctgcaccggc
actgactaaaagccagactgaccgtctggaggttctgctgaacccgaaagatgaaatcagcctgaactctggcaaaccgttccgtg
aactggaatccgaactgctgtctcgtcgtaagaaagacctgcaacaaatctatgctgaagagcgtgaaaactacctgggtaaactg
gaacgtgaaatcacccgtttctttgtggaccgtggtttcctggaaatcaagtctccgatcctgatcccgctggaatacatcgagcgcat
gggtattgataacgacaccgaactgtccaagcagattttccgtgtggacaagaacttctgcctgcgtccgatgatggcaccgaacct
gctgaatctggcgcgtaaactggatcgtgcactgccggacccgatcaaaatctttgaaatcggtccatgctatcgtaaggagagcga
cggtaaagaacacctggaagagttcactatgctgaactttttcagatgggttctggctgcacccgtgaaaatctggaatctatcatcac
cgacttcctgaaccacctgggcattgacttcaaaatcgttggtgattcctgcatggtttacggtgacactctggacgttatgcatggtgat
ctggaactgagcagcgctgttgtgggtccgattccgctggatcgtgaatggggtatcgataaaccgtggattggtgctggcttcggtct
ggaacgtctgctgaaagttaagcacgactttaagaacatcaaacgtgctgcgcgttccgagtcctattacaacggcattagcactaa
cctgtaagtcgaccatcatcatcatcatcattgagtttaaacggtctccagcttggctgttttggcggatgagagaagattttcagcctgat
acagattaaatcagaacgcagaagcggtctgataaaacagaatttgcctggcggcagtagcgcggtggtcccacctgaccccatg
ccgaactcagaagtgaaacgccgtagcgccgatggtagtgtggggtctccccatgcgagagtagggaactgccaggcatcaaat
aaaacgaaaggctcagtcgaaagactgggccttgtttgtgagctcccggtcatcaatcatccccataatccttgttagattatcaattta
aaaaactaacagttgtcagcctgtcccgctttaatatcatacgccgttatacgttgtttacgctttgaggaatcccatatgatacgcggcc
gctttcaaacgctaaattgcctgatgcgctacgcttatcaggcctacatgatctctgcaatatattgagtttgcgtgcttttgtaggccggat
aaggcgttcacgccgcatccggcaagaaacagcaaacaatccaaaacgccgcgttcagcggcgtttttctgcttttcttcgcgaatt
aattccgcttcgcaacatgtgagcaccggtttattgactaccggaagcagtgtgaccgtgtgcttctcaaatgcctgaggccagtttgct
caggctctccccgtggaggtaataattgacgatatgatcagtgcacggctaactaagcggcctgctgactttctcgccgatcaaaag
gcattttgctattaagggattgacgagggcgtatctgcgcagtaagatgcgccccgcattggaaacctgatcatgtagatcgaatgga
ctttaaatccgttcagccgggttagattcccggggtttccgccaaattcgaaaagcctgctcaacgagcaggctttttgcatgctcgag
cagctcagggtcgaatttgctttcgaatttctgccattcatccgcttattatcacttattcaggcgtagcaccaggcgtttaagggcacca
ataactgccttaaaaaaattacgccccgccctgccactcatcgcagtactgttgtaattcattaagcattctgccgacatggaagccat
cacagacggcatgatgaacctgaatcgccagcggcatcagcaccttgtcgccttgcgtataatatttgcccatggtgaaaacgggg
gcgaagaagttgtccatattggccacgtttaaatcaaaactggtgaaactcacccagggattggctgagacgaaaaacatattctca
ataaaccctttagggaaataggccaggttttcaccgtaacacgccacatcttgcgaatatatgtgtagaaactgccggaaatcgtcgt
ggtattcactccagagcgatgaaaacgtttcagtttgctcatggaaaacggtgtaacaagggtgaacactatcccatatcaccagctc
accgtctttcattgccatacggaattccggatgagcattcatcaggcgggcaagaatgtgaataaaggccggataaaacttgtgctta
tttttctttacggtctttaaaaaggccgtaatatccagctgaacggtctggttataggtacattgagcaactgactgaaatgcctcaaaat
gttctttacgatgccattgggatatatcaacggtggtatatccagtgatttttttctccattttagcttccttagc

Figure S8. Double incorporation plasmid maps for pBAD-sfGFP1TAG134TAA-CmaPylS/CmaPylT-C41AU and pEVOL-AcKRS1/MmPylT-UUA.

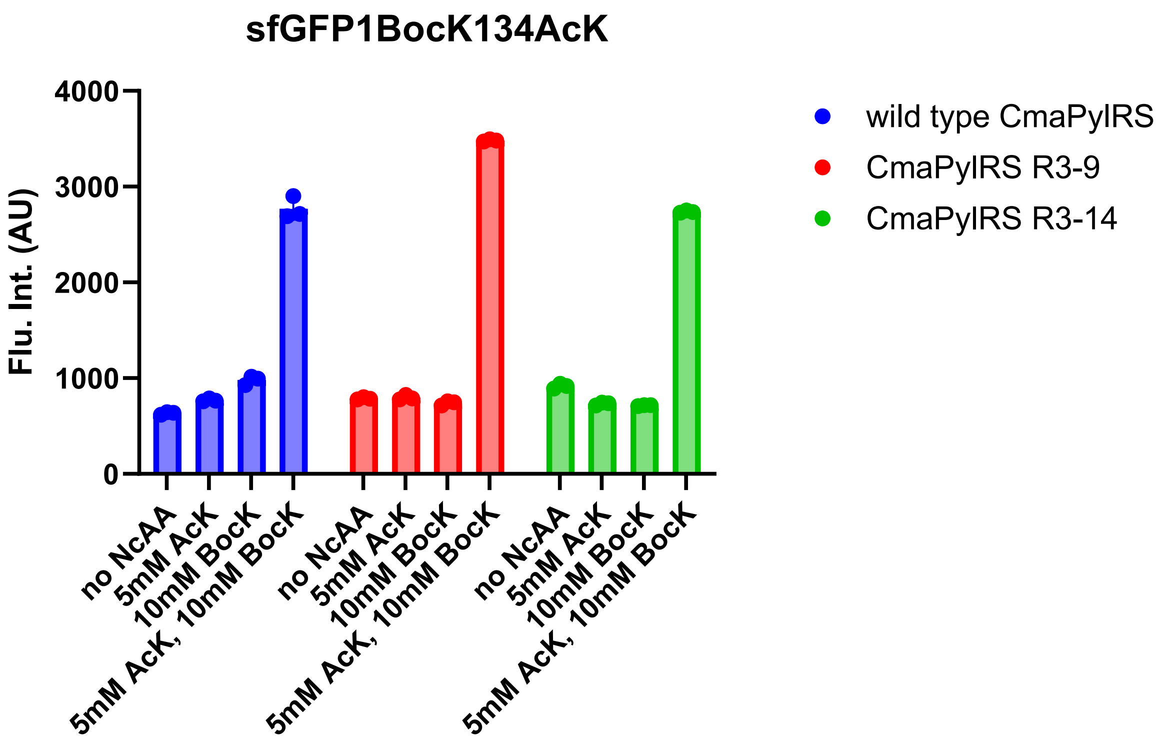

Figure S9 Expression of sfGFP1BocK134AcK with wild type CmaPylRS and evolved CmaPylRSs. With only added either 5 mM AcK or 10 mM BocK, there is little to no fluorescence compared to the background. One added both 5 mM AcK and 10 mM BocK expressed full length sfGFP.

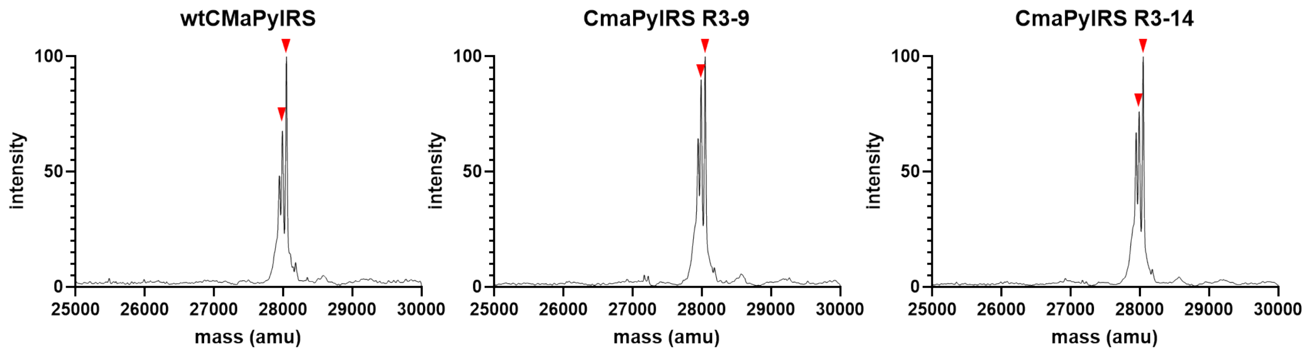

Figure S10. Deconvoluted ESI-MS spectra of purified sfGFP1BocK134AcK expressed using wtCmaPylS, CmaPylS-R3-9 or CmaPylS-R3-14. The labeled peaks are deconvoluted mass at 27,992 and 28,050 Da.
